# Hidden hunger in Europe: a review on determinants, fragmented policy responses, and implementation barriers

**DOI:** 10.3389/fnut.2025.1669008

**Published:** 2025-10-20

**Authors:** Alessandro L. Gallina, Suzan Otay, Jaisalmer de Frutos-Lucas, Marion Buso, Paula Moral Martinez, Kevin D. Cashman, Mairead E. Kiely, Siân Astley

**Affiliations:** ^1^European Public Health Alliance, Brussels, Belgium; ^2^EuroFIR AISBL, Brussels, Belgium; ^3^Cork Centre for Vitamin D and Nutrition Research, School of Food and Nutritional Sciences, University College Cork, Cork, Ireland; ^4^INFANT Maternal and Child Health Research Centre, University College Cork, Cork, Ireland

**Keywords:** micronutrient deficiencies, food fortification, hidden hunger, nutrition policy, determinants of health, zero hidden hunger EU

## Abstract

Despite significant improvements in food security and healthcare, micronutrient deficiencies – or “hidden hunger” – remain a widespread and under-recognized public health issue across Europe. These deficiencies impair metabolic, cognitive, and immune functions, and are linked to non-communicable diseases and increased morbidity in an aging European population. This policy review, undertaken within the European Union (EU)-funded Zero Hidden Hunger EU project, examines the demographic, socioeconomic, and geographical drivers of micronutrient deficiencies in Europe and evaluates existing national and EU-level policy responses. Vulnerable populations, including children, women of reproductive age, older adults, low-income households, and those living in low-UV regions, face disproportionately high risks due to intersecting biological, environmental, and social factors. The review also identifies substantial variation in national fortification strategies, revealing fragmented regulatory frameworks and inconsistent implementation. While EU legislation offers a harmonized structure for voluntary fortification, its flexibility has enabled broad national divergence, limiting the coherence and impact of public health efforts. Evidence from national policies illustrates both the potential and the shortcomings of current approaches. This review calls for more equity-oriented, mandatory, and evidence-based public health strategies, to address micronutrient malnutrition, including fortification, supported by comprehensive nutrition education and social protection measures. Initiatives such as the Zero Hidden Hunger EU project represent a critical opportunity for participatory policy co-creation, aiming to close data gaps and advance more inclusive and sustainable nutrition policies across Europe.

## 1 Introduction

The human body requires several essential compounds that cannot be synthesized endogenously, including 9 essential amino acids and 19 micronutrients - vitamins, trace elements, and minerals. These micronutrients are fundamental to health, influencing physical and mental development, immune competence, and regulation of metabolic processes. Importantly, many nutrients act in concert, meaning insufficiency in one can disrupt inter-dependent pathways, leading to non-specific, often overlooked symptoms (1). While clinical deficiencies such as scurvy (vitamin C), rickets (vitamin D), beriberi (vitamin B1), and pellagra (niacin) are well-documented, other micronutrients as well as insufficiency are less well studied but might also exert a significant influence on disease risk and long-term health (1). Deficiency symptoms rarely occur in isolation; they often reflect inadequacies in multiple nutrients, pointing to broader dietary insufficiencies. Increasing evidence links insufficiency to risk and progression of major non-communicable diseases (NCDs), such as cardiovascular disease, type 2 diabetes, osteoporosis, and certain cancers, underscoring the role of micronutrients in growth and development in children, as maintaining health and reducing risk of NCDs among adults (2).

More than 2 billion people worldwide are affected by micronutrient deficiencies (3). Although such deficiencies are commonly associated with low-income settings, they also persist in high- and middle-income regions, including Europe, where undernutrition often manifests not as hunger but a chronic lack of essential micronutrients, termed “hidden hunger,” and coexists with overnutrition, characterized by overweight and obesity (4). Historically, Europe witnessed a dramatic reduction in overt nutritional deficiencies due to enhanced food availability and improved healthcare access (5, 6); however, the prevalence of subclinical deficiencies remained widespread yet often unnoticed (6–8). Hidden hunger undercuts public health by increasing the risk of morbidity and mortality, weakening immune function, and impairing physical and mental growth and development (6). Even individuals who appear well-nourished experience hidden hunger, highlighting the limitations of using caloric intake or body weight as indicators of nutritional adequacy (1). At the population level, micronutrient deficiencies contribute to substantial productivity losses and economic burdens due to increased healthcare utilization and reduced workforce participation (9).

This review mapped and analyzed national and European Union (EU)-level policies related to micronutrient intake, deficiencies, and health status, with three primary objectives: (1) to understand the main factors driving micronutrient deficiencies in Europe; (2) to evaluate existing policy responses; and (3) to identify barriers limiting their effectiveness.

### 1.1 Determinants of hidden hunger

#### 1.1.1 Demographic factors

Demographic factors profoundly affect micronutrient requirements and dietary patterns, contributing to observed disparities in nutritional status across the WHO European Region (53 countries), the EU (27 countries), the European Economic Area (EEA – 30 countries), and the World Bank’s Europe and Central Asia (ECA) region (28 countries) (10). Age and sex profoundly influence micronutrient requirements, dietary habits, and vulnerability to deficiencies across populations in Europe. Across the life course, specific physiological and developmental stages influence nutritional needs. Infants, children, and adolescents require elevated nutritional needs to support rapid physical and cognitive development. However, contemporary European diets, often high in energy but low in nutritional quality, can fail to meet these increased requirements (11). In Europe, common deficiencies include iodine, iron and vitamin D; iodine deficiency during childhood can result in irreversible intellectual impairment (12) and is considered the most preventable cause of brain damage in the fetus and infant (2, 13); iron deficiency in early life is associated with impaired cognitive function, poor academic achievement, stunted growth, and weakened immune responses (2), while low vitamin D increases risk of rickets and compromised immunity (6).

Women of reproductive age have increased micronutrient demands compared to men, primarily due to menstruation, pregnancy, and lactation. In 2019, 14% of females aged 15–49 in the EU were anemic (14), with prevalence rising by 4.1 percentage points between 2005 and 2016 (8). Folate deficiency, despite long-standing public health guidance, remains common and contributes to neural tube defects (NTDs) such as spina bifida and anencephaly (6), and cuts across socioeconomic boundaries even in high-income countries (15).

In later life, middle-aged and older adults often experience diminished appetite, altered taste and smell, medication interactions, and impaired nutrient absorption, contributing to deficiencies in several key micronutrients (2). Calcium and vitamin D are essential for maintaining bone health and preventing osteoporosis and osteomalacia. Deficiencies in both substantially increase the risk of falls, fractures, and long-term dependency (6, 16). Folate and vitamin B12 are linked to cognitive function, and low intakes and absorption are associated with increased memory loss, and risk of dementia and cardiovascular disease (2). Together, these factors highlight the need for tailored nutrition strategies for an aging population across Europe.

Demographic factors are interlinked and often exert effects across generations. Maternal undernutrition and micronutrient deficiencies can negatively affect pregnancy outcomes and fetal development, creating a cycle of poor health. For instance, inadequate calcium intake during pregnancy is associated with preeclampsia, low birth weight, and reduced bone development in the fetus (17). Similarly, deficiencies in iodine during pregnancy can harm both mother and child, affecting fetal brain development and increasing risks of neurodevelopmental disorders (1). Zinc deficiency has also been linked to adverse pregnancy outcomes, including intrauterine growth restriction and complications during labor (2). Early-life micronutrient deficiencies can affect educational attainment, employment prospects, and long-term resilience, with effects persisting across a (reduced) lifespan. For women, suboptimal bone mineral density established in adolescence or exacerbated during menopause increases the likelihood of osteoporosis and fractures in mid- and older age (18). These linkages highlight how risks are not isolated but cascade and amplify across the lifespan.

Sex further shapes risk of micronutrient deficiencies across the life course. Even in high-income settings, females are disproportionately affected by iron deficiency and anemia (19), and these risks persist into later life (20). Calcium deficiency is also more common in females, contributing to higher rates of osteoporosis and fracture. Globally, hip fractures are expected to increase from 1.7 million in 1990 to 6.3 million by 2050, with females comprising 80% of cases. Lifetime risk of osteoporotic fracture in females is estimated at 30–40%, compared to 13% in males (2). These data highlight that sex- and age-related disparities in micronutrient status are often under-recognized, despite their significant long-term health consequences.

#### 1.1.2 Socioeconomic, ethnic, and geographical factors

Socioeconomic status is a major determinant of dietary quality and micronutrient intake. Across Europe, low-income individuals and households face greater challenges in accessing nutrient dense foods, such as fruits, vegetables, whole grains, and lean proteins. Nutrient-rich foods tend to be more expensive per calorie than energy-dense, nutrient-poor alternatives (1). As a result, economically disadvantaged groups including single-parent families, migrants, and unemployed individuals can be forced to prioritize energy intake over nutrient density, leading to diets that are calorically adequate but micronutrient deficient (1). This dynamic contributes to a well-documented vicious cycle of poverty, malnutrition, and disease – a pattern observed in all countries, regardless of income level, with varying degrees of intensity. In both low- and high-income settings, economic constraints push vulnerable groups toward diets dominated by affordable staples like refined grains, which may alleviate hunger but fail to meet essential micronutrient requirements (1). In EU countries, while the cheapest food options for lower-income households are not exclusively grains, they frequently include low-priced, low-quality meat. These diets, despite potentially contributing to overweight and obesity, often lack essential micronutrients (1).

Across the ECA region, household food expenditure varies widely, from 7 to 66%, reflecting income inequality and purchasing power disparities (21). Low-income households frequently rely on inexpensive foods (e.g., refined flour, starchy vegetables, processed meats) that are high in sodium (salt) but low in micronutrients (21). Systematic reviews show that lower socioeconomic groups in Europe consistently consume fewer micronutrients, including iron, than more affluent counterparts. Although methodological differences make cross-study comparisons difficult, consistent evidence points to inadequate intakes of B vitamins, folate, zinc, and iron among lower-income populations (22, 23). The WHO European Childhood Obesity Surveillance Initiative found that children aged 6–9 from families with lower socioeconomic status consumed fewer fruits and vegetables than those from higher-status families (24); a concerning trend given these foods are key sources of vitamins A and C in children’s diets (25).

Geographical variation across Europe further shapes dietary patterns and micronutrient adequacy. Vitamin D status, for example, is heavily influenced by latitude and seasonal sun exposure (26). In northern countries, public health authorities frequently recommend vitamin D supplementation for vulnerable groups, including young children and the elderly. While fortification and supplementation policies have helped reduce deficiencies, debates continue regarding optimal intakes and the balance between dietary sources and UV exposure (27). Regional dietary patterns also influence nutrient intake. Mediterranean countries such as Greece and Italy typically exhibit higher vitamin E intakes but lower vitamin D and retinol, partly due to dietary preferences and limited fortification. In contrast, Nordic countries have higher intakes of vitamin D and retinol due to effective food fortification policies and supplement use (28).

### 1.2 Approaches to tackle micronutrient deficiencies

The recognition of micronutrient deficiencies as a public health challenge dates back millennia, with early interventions rooted in empirical observations. Ancient Egyptians prescribed liver - rich in vitamin A - to treat nyctalopia or “night blindness” (29), while 18th-century sailors consumed citrus fruits to treat scurvy (1). These early therapeutic approaches laid foundations for modern micronutrient policy, which has evolved from treating deficiencies to preventing suboptimal intakes (1). Today, public health interventions typically follow four core approaches: food fortification, supplementation, nutrition education to encourage diverse, high-quality diets, and broader public health measures such as infection control (2). Successful implementation requires coordinated action across sectors, including health, agriculture, education, social policy, economic development, and both governmental and non-governmental actors. Reflecting this historical trajectory, the present review adopts a chronological and evolutionary perspective, examining how interventions have shifted from individual, therapeutic, and nationally driven actions to preventive, population-level, and increasingly EU-coordinated frameworks.

Micronutrient supplementation involves providing vitamins and minerals, often in the form of capsules, powders, or syrups, to correct specific deficiencies (7). In Europe, supplementation is typically based on clinical guidelines or public health recommendations rather than mandatory policy. For instance, vitamin D is recommended for infants from the first week of life in Nordic countries due to low concentrations in breast milk and limited sun exposure (30). Folic acid is also advised before and during pregnancy to prevent neural tube defects (31). While supplementation can be effective, challenges persist around sustainability, adherence, and access, particularly among vulnerable groups (32), and supplements cannot fully substitute for broad nutrient diversity found in a healthy, balanced diet (7).

Food fortification, a complementary and widely adopted preventive strategy, entails adding one or more micronutrients to food products to enhance nutritional quality (1). With rising urbanization and increased consumption of poor-quality low nutrient-dense diets that are high in energy-dense foods of little nutritional value, fortification offers an efficient way to reach large segments of the population, including marginalized groups. However, effective fortification requires strong regulatory frameworks, evidence-based planning, continuous monitoring of intake and food safety, and enforcement of nutrient standards. Public communication and social marketing are also essential to build consumer trust and ensure uptake (6).

Together, these approaches illustrate both the progress and the ongoing complexity of tackling micronutrient deficiencies in Europe. Despite evolving strategies, persistent inequities and multifactorial drivers of hidden hunger continue to limit the effectiveness of interventions. These deficiencies are not only shaped by geography, socioeconomic status, or sex, but also carry intergenerational consequences, affecting fetal development, childhood growth, and long-term cognitive and physical health outcomes. These challenges underscore the need for equity-sensitive approaches that address not only biological needs, but also structural barriers to access, affordability, and participation. A clearer understanding of national and regional policy efforts is urgently needed to identify successful models, address gaps, and support more equitable, evidence-based, and harmonized action across the European context.

## 2 Methods

### 2.1 Study design

This policy review was conducted as part of the *Zero Hidden Hunger EU* project, funded under the EU’s Horizon Europe research and innovation program (Grant Agreement No. 101137127). The key objective was to systematically identify, map, and analyze policies and strategic documents related to micronutrient intake, deficiency, and health status across Europe. The review aimed to shed light on (1) the primary factors impacting micronutrient deficiencies in Europe, (2) the policy-based responses implemented across the region, and (3) the barriers limiting the effectiveness of these interventions.

The review followed a predefined protocol developed collaboratively by project partners and published prior to the search phase of the review on Zenodo (33), providing a transparent and pre-registered methodological framework. This protocol informed all stages of the review, including the search strategy, eligibility criteria, and data synthesis approach.

### 2.2 Knowledge gap

Several reviews have examined micronutrient intake and nutritional status across Europe, exploring the influence of socio-economic determinants (23), methodological inconsistencies in assessing intake adequacy (34), and the contribution of voluntary food fortification to nutrient intakes and biomarker status (35). While these analyses acknowledge the role of regulatory variation, their primary emphasis remains on nutritional outcomes rather than the policy instruments themselves. To our knowledge, no previous review has systematically mapped and analyzed national- and EU-level policies specifically targeting micronutrient deficiencies. This review addresses that gap by focusing exclusively on policy frameworks, their implementation modalities, and the barriers limiting their effectiveness.

### 2.3 Search strategy

A structured one core search was conducted between January and April 2025 to identify relevant policy documents published between 1995 and 2024. The search included both academic and grey literature sources. The databases and platforms searched were: PubMed, OpenGrey, the European Food Safety Authority (EFSA), the Food and Agriculture Organization (FAO), the World Health Organization (WHO) and WHO’s Global database on the Implementation of Food and Nutrition Action (GIFNA), the Micronutrient Forum (MNF), the Global Alliance for Improved Nutrition (GAIN), Nutrition International, World Bank, and the Publications Office of the EU. Database-specific filters were applied where available. The GIFNA database was searched using nutrient-specific and regional filters to identify national policies within the WHO European Region. Due to limitations in search functionality, some repositories required manual iterative searches. In particular, the search of the Publications Office of the EU posed significant challenges, as its database does not support advanced search functionalities such as parameter filters or structured queries. Despite the temporal window defined in the inclusion criteria, a small number of pre-1995 sources were retrieved *ad hoc* and included in the narrative to provide historical context, particularly to illustrate early policy adoption in certain countries. These references help trace the evolution of fortification policies and approaches.

Search terms were grouped into four conceptual categories: nutrient-related terms, descriptors of nutritional status, policy terms, and geographic filter. The conceptual categories and corresponding search terms are summarized in Table 1.

**Table 1:** Search terms.

Category	Search terms used
Nutrient-related terms	Micronutrient, micronutrients, vitamin, vitamins, mineral, minerals
Descriptors of nutritional status	Deficiency, deficiencies, inadequacy, inadequacies, insufficiency, insufficiencies, consumption, fortification, supplementation, intake, requirements, standard, standards
Policy terms	Guideline, guidelines, policy, policies, program, programs, regulation, regulations
Geographic filter	Europe

Boolean operators and truncations (e.g., *deficien* for *deficiency* and *deficiencies*) were used to enhance sensitivity. For institutional websites without advanced search tools, targeted Google searches, restricted to PDF file types, were applied and scraping tools such as *ImportFromWeb* (36) were used to support document retrieval.

### 2.4 Record identification summary

A total of 1,648 records were retrieved across all sources, including 160 from PubMed, 300 from the WHO (298 and 2 manual searches) and 145 from WHO’s GIFNA, 299 from the FAO, 281 from the EFSA, 25 from the MNF, 102 from GAIN, 46 from Nutrition International, and 275 from the World Bank. Due to the lack of advanced search functionalities and parameter filters in the Publications Office of the EU database, the identification of relevant policy documents was challenging and required manual screening. These limitations in search precision, combined with a high volume of “out of scope” results, may have affected the comprehensiveness of the retrieval. To enhance specificity, a set of predefined terms was applied in the manual screening process, including: *“fortification,” “supplementation,” “micronutrient supplementation,” “hidden hunger,” “micronutrient deficiency,”* and *“vitamin deficiency.”* Similarly, the OpenGrey database was not searched in its entirety. Instead, a manual review was performed within the “Life Sciences” category to identify pertinent records.

### 2.5 Eligibility criteria

All records retrieved were screened for their relevance. Documents were eligible for inclusion if they were published between January 1995 and April 2024, focused on countries within the broader European region, including the EU, the WHO European Region, or the World Bank’s ECA region, and addressed micronutrient deficiencies or related interventions through public health policy or regulation. To ensure accessibility and comparability, only documents available in English or with English translations were considered. Eligible sources included those issued by governmental, academic, or intergovernmental institutions. Documents were excluded if they originated from private or commercial entities with potential conflicts of interest; focused on non-human health contexts such as feedstuffs; animal or *in vitro* studies; or addressed acute conditions like famine and starvation not directly linked to micronutrient policy. Sources that focused on populations suffering from specific pathologies not directly related to micronutrient deficiencies were also excluded. Only policy-oriented documents were included, with primary research sources excluded.

Grey literature (e.g., national strategy documents) was included if it originated from governmental, intergovernmental, or recognized public health institutions. Materials were assessed based on issuing authority, geographic scope, and thematic relevance to hidden hunger, ensuring that only policy-relevant documents were retained. No documents from commercial actors or industry-affiliated bodies were included.

All documents retrieved from PubMed were imported into Rayyan, a web-based tool for systematic reviews (37). The screening process began with titles and abstracts, followed by full-text assessment to determine final eligibility.

This screening process resulted in the inclusion of 318 final documents for full eligibility assessment. After a second and more thorough assessment, 129 documents were preliminary selected for inclusion. However, many of these documents had a global or regional scope, whereas our subsequent analytical focus required country-specific data. To facilitate meaningful comparisons across national contexts, we refined our inclusion criteria to prioritize documents with a clear country-level perspective. Additionally, while our initial retrieval included national strategic plans, guidelines, and recommendations, we ultimately limited our analysis to formal policy documents, in line with the defined scope of the study.

### 2.6 Supplementary searches

**Figure 1:**
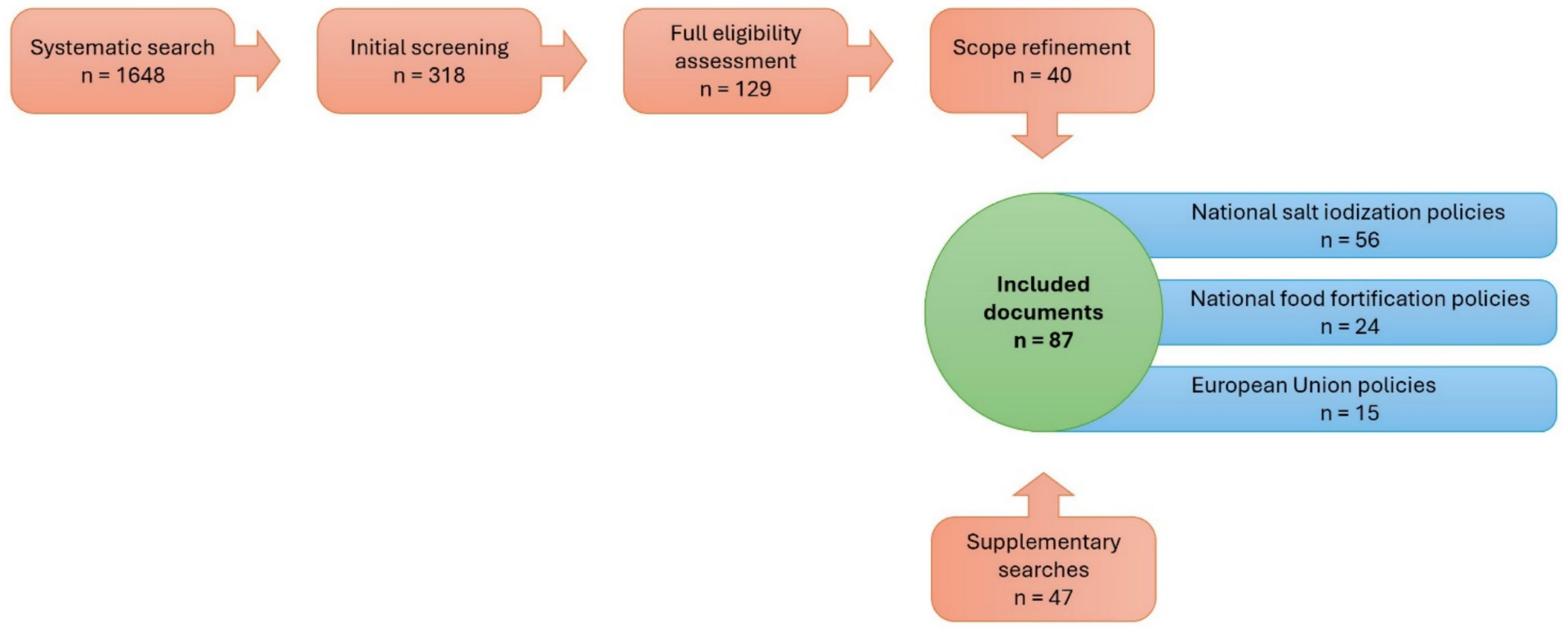
Flow diagram of the document identification, screening, and inclusion process: The figure illustrates the sequential steps of the policy review, beginning with the systematic search (*n* = 1,648), followed by initial screening (*n* = 318), full eligibility assessment (*n* = 129), and scope refinement (*n* = 40). An additional 47 documents were identified through supplementary searches. The final dataset comprised 87 included documents, categorized into national salt iodization policies (*n* = 56), national food fortification policies (*n* = 24), and European Union policies (*n* = 15). The numbers across categories do not add up to the total because some documents were relevant to more than one category and therefore included in multiple groups.

Following the initial structured search, an additional search was conducted on the Global Fortification Data Exchange interactive website, following the same criteria described in the protocol published on Zenodo (33). Moreover, a number of relevant country-level policies were found to be missing or underrepresented. To address these gaps, additional *ad hoc* searches were conducted for all the countries of the EEA region, including the UK. This included targeted reviews of national government websites, ministries of health, and nutrition strategy documents. Since at this stage some relevant sources were identified that were not available in English, where necessary, documents were translated using a combination of machine translation and expert verification. These complementary identified sources were assessed and included based on the same eligibility criteria as for the main search (except for the language criterion, as indicated above). This led to the incorporation of 47 additional relevant documents, 31 national salt iodization policies and 16 national food fortification policies. In total, 87 documents were selected through a combination of systematic and supplementary searches. The complete selection and screening process is visualized in Figure 1. Because certain documents were relevant to more than one policy category, they were assigned to multiple groups. Consequently, the sum of documents across categories exceeds the total number of unique documents.

### 2.7 Data extraction

For each document included, data were extracted using a standardized template that captured key information such as the title and issuing organization, year of publication, geographic focus, and the specific micronutrient(s) and population group(s) targeted. The template also recorded the type and scope of the policy instrument, such as strategic plans, regulations, or guidelines, along with its stated objectives, proposed interventions, reported health outcomes, and, where available, information on implementation status. To ensure comparability across sources, all micronutrient values were standardized to micrograms (μg) and milligrams (mg).

### 2.8 Data synthesis and visualization

Thematic analysis of the included documents was conducted manually. Initial reviewers screened and categorized records as “yes,” “tentative,” or “no” for inclusion; after which, different reviewers reassessed all “yes” and “tentative” records and determined the final set. Categories were developed inductively during review rather than from a fixed coding framework. Policies were categorized by type (e.g., mandatory fortification, voluntary guidelines), geographic scope (national vs. EU-level), and target populations at-risk of hidden hunger (e.g., children, women of reproductive age). Cross-cutting themes, gaps, and regional patterns were identified and synthesized narratively.

To visualize summarizing the document identification and screening process, a flowchart was presented, created using Affinity Designer (version 1.10.6.665, Serif Europe Ltd). To support the analysis all included policies were listed and organized in structured tables using Microsoft Excel for Microsoft 365 (38). A map illustrating differences in salt iodization legislation across Europe was created using MapChart (39) to visually illustrate policy variations across the region. This work is licensed under a Creative Commons Attribution-ShareAlike 4.0 International License.

## 3 Results

Of the 1,425 records retrieved from all sources, 172 were deemed suitable for analysis and categorized as follows: 41 national regulations, 15 national strategic plans, 12 EU regulations and directives, 27 scientific opinions, 6 EU strategic plans, 5 parliamentary questions, 15 national guidelines and recommendations, 16 guidelines and recommendations for Europe, and 16 scientific articles. An additional 19 records, while not classified into these categories, were retained due to their relevance to the topic and their contribution to the background and introduction of the review. Using this collection as the basis, following subsections will provide a summary of national and EU-level interventions across Europe aimed at addressing micronutrient deficiencies, with a focus on the historical evolution of such policies. In particular, this review focuses explicitly on food-based micronutrient strategies, particularly food fortification. Although supplementation recommendations represent another crucial public health measure to address deficiencies, especially in high-risk or vulnerable populations, they typically appear in public health guidelines rather than binding legislation and thus are not systematically mapped here.

This analysis begins by assessing the coverage and approaches of key national micronutrient fortification policies, including salt iodization and other food fortification measures. It traces the historical development of these policies in selected countries and across Europe, highlighting how they have evolved in response to emerging public health needs. The review then explores the variations in policy implementation across different European regions, emphasizing disparities in regulatory frameworks and strategies.

### 3.1 National food fortification policies in Europe

The fortification of food with micronutrients has emerged as a cornerstone of population-level public health strategies (6). Its development is rooted in early scientific discoveries in the 19th century linking the use of certain foods to treat vitamin deficiency related diseases, such as the use of cod liver oil (vitamin D) to cure rickets or rice bran (vitamin B_1_) to treat beriberi (5). These findings laid the groundwork for systematic food-based micronutrient interventions. In the 20th century in Europe, oils and fats began to be fortified with vitamins A and D to address xerophthalmia and rickets, while cereal products were enriched with iron and B-vitamins (1). The addition of folic acid to flour in industrialized countries has led to dramatic reductions in NTDs (40). Today, food fortification is promoted not only for its historical successes but also for its preventive potential, cost-effectiveness, and sustainability (6). By leveraging existing food systems, it enhances the nutritional quality of widely consumed foods without requiring behavior change, achieving high coverage and helping reduce the prevalence of a plethora of diet-related conditions (6).

Policies shape how fortification and other micronutrient interventions are implemented. They can take mandatory, voluntary, or targeted forms. Mandatory policies establish legal frameworks requiring fortification or supplementation to secure widespread compliance and maximize public health impact. Voluntary approaches encourage but do not oblige the addition of micronutrients, relying instead on market incentives and consumer demand. Targeted policies address the needs of vulnerable or high-risk groups, such as pregnant women or children, through specially formulated products. Meanwhile, small-scale community-based interventions tackle localized deficiencies through pilot or grassroots projects. Together, these policies and strategies complement one another to address micronutrient deficiencies at both population and community levels (2).

#### 3.1.1 National salt iodization policies for countries in the wider European region

One of the most successful examples is salt iodization, which represents the first large-scale micronutrient fortification policy in modern history (6). Introduced in Switzerland in 1922 to prevent goiter and congenital hypothyroidism, iodine fortification proved highly effective, with congenital hypothyroidism disappearing by 1930 and a marked decline in goiter prevalence among schoolchildren (41, 42). In 1993, the WHO and UNICEF endorsed universal salt iodization as the primary global strategy to eliminate iodine deficiency disorders (43). This success catalyzed the global spread of salt iodization, yet Europe’s current patchwork of iodization policies remains fragmented. The comprehensive 2024 WHO report *Prevention and control of iodine deficiency in the WHO European Region* (44), which analyses legislation, frameworks, implementation, and coverage rates across Europe, provided an insightful overview for the 27 EU Member States, the three additional countries in the EEA region, Iceland, Liechtenstein, and Norway, as well as the United Kingdom (UK). The latter was included in our review in light of the fact that many of its current national policies on food fortification were developed prior to Brexit and continue to reflect EU-aligned standards in several areas.

**Figure 2:**
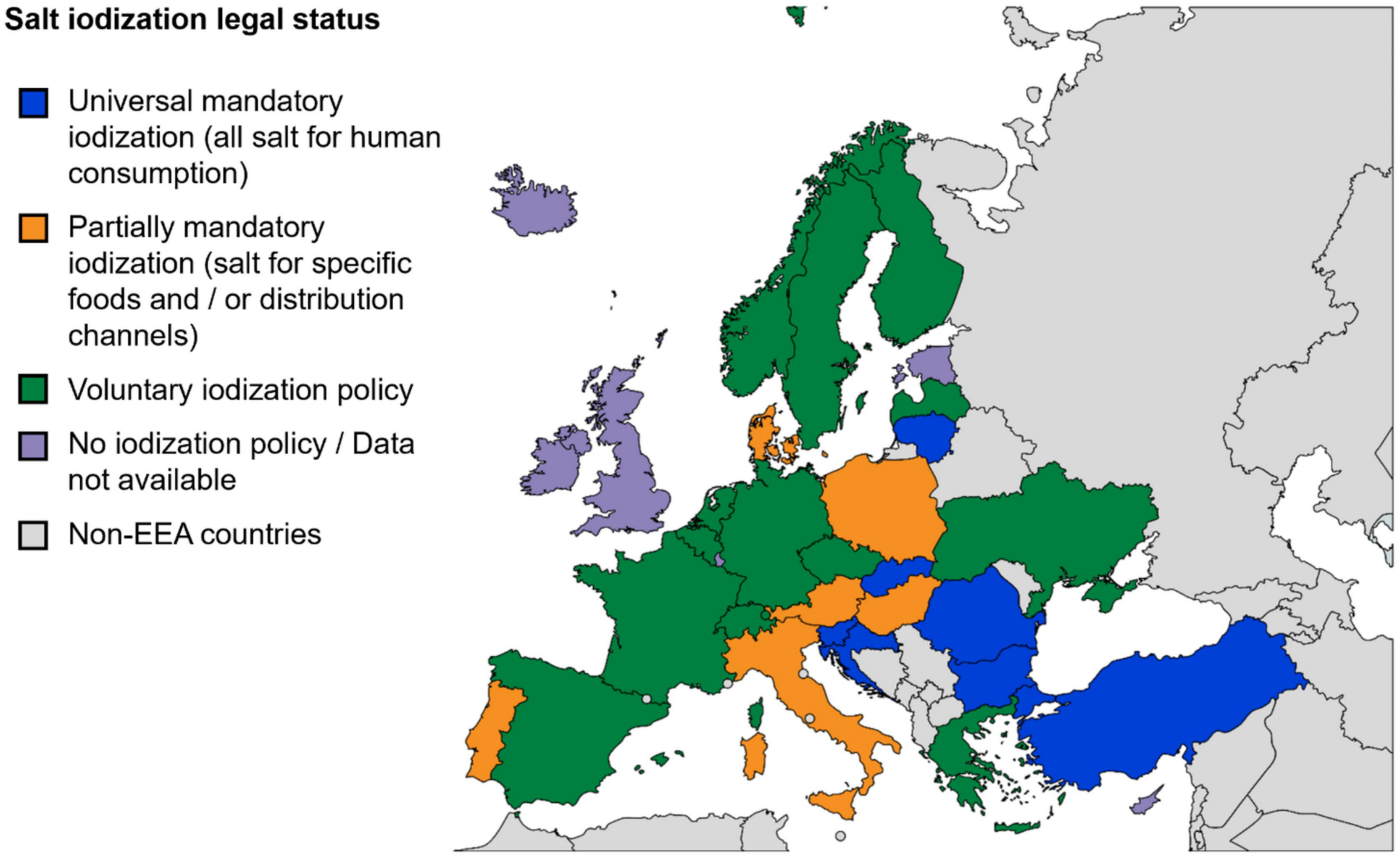
Salt iodization policies across the EEA and UK: Legal frameworks for salt iodization vary widely across the region, ranging from universal mandatory to voluntary or absent policies. This map, reflecting the findings of Ref. (44) and cross-checked against the sources listed in Supplementary Table 1, illustrates the persistent regulatory fragmentation in iodine deficiency prevention in Europe.

Salt iodization policies between these countries remain highly fragmented and vary significantly, ranging from mandatory to voluntary fortification (see Figure 2), with mandatory laws typically specifying precise iodine content ranges, commonly 15–55 mg/kg salt, and permitted compounds, primarily potassium iodide (KI) or potassium iodate (KIO₃); sodium iodide (NaI) and sodium iodate (NaIO₃) are permitted less frequently (see Supplementary Table 1). This regulatory diversity is evident even among nations with long-standing mandatory programs, where standards have evolved through decades of legislative refinement. Among the 33 countries analyzed in this review, 7 enforce universal mandatory iodization for all salt intended for human consumption (Bulgaria, Croatia, Lithuania, Romania, Slovakia, Slovenia, Turkey). In addition to these, 6 countries (Austria, Denmark, Hungary, Italy, Poland, Portugal) have adopted partial mandatory policies: bakery salt (Austria), household and bakery salt (Denmark), mass catering (Hungary), default retail sale (Italy), table salt only (Poland), and school meals (Portugal). The remaining 20 countries operate under voluntary regimes (Belgium, Czechia, Finland, France, Germany, Greece, Latvia, Liechtenstein, Netherlands, Norway, Spain, Sweden, Switzerland, Ukraine) or lack formal policies (Cyprus, Estonia, Iceland, Ireland, Luxembourg, UK) (see Figure 2). Notably, voluntary policies are more common in Western Europe, while mandatory frameworks tend to cluster in Eastern and Central Europe.

The complete overview of specific national legislation and detailed iodine content standards is provided in Supplementary Table 1, which shows that only a minority of countries have adopted universal mandatory iodization - an approach generally associated with higher household coverage and improved iodine status (44). The majority of countries, however, continue to rely on partial, voluntary, or absent frameworks, contributing to fragmented implementation across the region.

#### 3.1.2 National food fortification policies in Europe beyond iodine

A review of food fortification policies, beyond iodine, in EEA countries demonstrates diverse approaches to addressing micronutrient deficiencies. Among the 15 EEA countries with detailed national-level food fortification policies, six (Austria, Belgium, Finland, Poland, Sweden, and the UK) enforce mandatory fortification policies targeting specific food vehicles (see Supplementary Table 2). The remaining nine EEA countries (Germany, Greece, Hungary, Ireland, the Netherlands, Norway, Liechtenstein, Switzerland, and Turkey) implement voluntary but regulated fortification schemes.

These policies reflect diverse nutritional priorities, health goals, and regulatory approaches. Mandatory fortification policies, such as those in Austria, Belgium, Finland, Poland, Sweden, and the UK, typically target fortification of specific food vehicles with specific micronutrients to address recognized public health needs. For example, Belgium’s mandatory fortification of margarines and edible fats with vitamins A and D, implemented since 1980, is an exemplar of addressing widespread micronutrient deficiencies through staple foods. Similarly, Austria enforces mandatory fortification of infant formula and follow-on formulas with critical nutrients, notably vitamin D and iron, demonstrating a targeted approach aimed at vulnerable populations like infants. Sweden and Finland also exhibit targeted mandatory fortification policies, focusing predominantly on vitamin D to mitigate deficiencies in northern European populations. Building on its previous voluntary, albeit widely practiced fortification regulations, Finland mandated for vitamin D fortification of skimmed homogenized milk, reflecting dietary patterns and consumption habits. Sweden’s comprehensive vitamin D fortification policy covers milk products, margarine, and plant-based drinks, applying clearly defined nutrient ranges to achieve consistent public health outcomes.

Voluntary fortification, though more-widely adopted, varies considerably across Europe (see Supplementary Table 2), with countries like Germany, Greece, Hungary, Ireland, the Netherlands, and Norway, employing distinct regulatory frameworks. Germany and Greece allow voluntary fortification across selected foods such as cereals, dairy, beverages, and confectionery, but the fortification of these foods is regulated carefully either through explicit nutrient limits or case-by-case approvals. The voluntary fortification of bread in Hungary and Ireland primarily addresses folic acid deficiencies, a measure reflecting strategic public health intent albeit without mandatory enforcement. In contrast, countries such as Liechtenstein, Switzerland, and Turkey permit broader voluntary fortification across all processed foodstuffs, albeit within rigorously defined maximum allowed levels. Turkey, notably, emphasizes that added nutrients must be bioavailable and serve clear public health objectives, indicating a structured yet flexible approach to voluntary fortification. The UK’s mandatory fortification policies for wheat flour, including the forthcoming addition of folic acid in 2026, illustrate proactive steps to address persistent micronutrient deficiencies within specific demographic groups. The clearly stipulated nutrient values reflect careful calibration based on evidence-driven health priorities.

Interestingly, of the 28 European countries on the EEA, 19 (Bulgaria, Croatia, Cyprus, Czech Republic, Denmark, Estonia, France, Iceland, Italy, Latvia, Lithuania, Luxembourg, Malta, Portugal, Romania, Slovakia, Slovenia, Spain, and Ukraine) lack specific national fortification policies beyond the overarching EU voluntary fortification framework (Regulation EC No 1925/2006 (45); discussed in the following section). This absence of explicit national regulations might limit the potential effectiveness and equity of fortification interventions in addressing public health nutrition needs across these populations. Further details around these national food fortification policies with various micronutrients are detailed in Supplementary Table 2.

Across the EEA region, food fortification policies most commonly target vitamins D, A, and folic acid, as well as key minerals and trace elements such as calcium, iodine, and iron. Vitamin D emerges as the most frequently mandated micronutrient, particularly in northern countries like Sweden and Finland, reflecting concerns over lack of dermal synthesis of vitamin D due to limited UVB-rich sunlight during the extended winter periods. Folic acid fortification, whether mandatory or voluntary, frequently appears in policies aiming to reduce NTDs, especially in bread and flour. Iron and calcium are also recurrently addressed, particularly in flour regulations such as those in the UK. Overall, while a few countries implement broad, mandatory policies, the dominant approach across the EEA is regulated voluntary fortification, tailored to national dietary patterns and public health needs.

##### 3.1.2.1 The evolution of vitamin D fortification policies in Finland

The Finnish experience over the past 10 years is exemplary and responsive with respect to not only implementation, but also evaluation of vitamin D fortification policies. This has been overviewed in more detail elsewhere (46), but in brief: the regulation of vitamin D fortification in Finland has evolved over several decades. The earliest legal basis can be traced back to Decree 182/1987 which regulated the vitamin D content in margarine, butter-vegetable oil mixtures, and fat mixtures, allowing voluntary fortification of these products with 5–10 μg vitamin D/100 g (47).

A major policy shift took place in 2002 with the Decree of the Ministry of Trade and Industry (917/2002), which expanded voluntary fortification to include all liquid dairy products (up to 0.5 μg vitamin D per 100 mL) and all fat spreads except butter (up to 10 μg vitamin D per 100 g) (48). However, data from the FINDIET 2007 Survey showed that average vitamin D intake remained below recommended levels among both men and women (49). In response, the Finnish National Nutrition Council, operating under the Finnish Food Authority, updated its recommendations in April 2010, doubling the suggested fortification levels to 1 μg per 100 mL for liquid dairy products (excluding organic milk) and 20 μg per 100 g for spreadable fats (49). Though not legally mandatory, these recommendations were widely adopted by the food industry (50) and, alongside increased supplement use, led to notable public health improvements. A prospective study demonstrated substantial increases in standardized serum 25-hydroxyvitamin D concentrations between 2000 and 2011 in both men and women. Among supplement non-users, those who regularly consumed fortified dairy products showed significantly greater increases in vitamin D status than non-consumers (51).

In 2016, Finland strengthened its vitamin D fortification policy through Ministry of Agriculture and Forestry Decree 754/2016 (52). This decree mandates that all homogenized, fat-free milk (≤0.5% fat) sold in Finland be fortified with vitamin D, which is fat soluble (53), at a minimum level of 1 μg per 100 mL, ensuring equitable vitamin D intake regardless of milk fat content. The FINDIET 2017 survey, which captured data after the new mandate, showed that Finnish adults’ vitamin D intake is well above previous levels, with only ~4% of adults fell below the 30 nmol/L deficiency threshold, and an estimated 77–91% of the Finnish population had serum levels in the sufficient range (≥50 nmol/L) depending on the subgroup (54). This example underscores the dynamic and adaptive nature of fortification policy when grounded in public health monitoring and evaluation.

### 3.2 EU regulatory frameworks for micronutrient fortification

In Europe, national legislations regarding food fortification differ considerably, reflecting diverse public health priorities, historical practices, and socio-economic contexts across individual countries. To address these disparities and ensure both consumer safety and market consistency, the EU has progressively developed harmonized frameworks through directives and regulations since the mid-1990s, as detailed chronologically in Table 2. EU laws standardize the addition of vitamins and minerals to foods across Member States and the European Economic Area (EEA), facilitating the functioning of the single market and ensuring nutritional adequacy and consumer safety.

**Table 2:** EU legislation relevant to micronutrient fortification.

Legislation	Year	Focus	Purpose
Delegated Regulation (EU) 2021/571 (68) (IF Formula Additives)	2021	Infant formula additives	Authorizes specific vitamin and mineral substances in infant formula and follow-on formula (including hydrolysate-based). It amends the annex of 609/2013 to add new approved nutrient sources for formula. This regulation ensures formula manufacturers may include EFSA‑evaluated micronutrient forms, maintaining infant nutrition standards.
Delegated Regulation (EU) 2017/1091 (69) (Baby Foods & FSMP Additives)	2017	Cereal-based baby foods and FSMP	Specifies which vitamin and mineral substances (and their chemical forms) may be added to processed cereal-based baby foods and to FSMP for infants. Amending the annex of Regulation 609/2013, it authorizes additional micronutrient sources for these products. This ensures that fortification of weaning foods and infant dietetic foods uses only approved nutrient forms.
Commission Regulation (EU) No 2017/1203 (70)	2017	Fortified foods and supplements (new sources)	Amends Directive 2002/46 and Regulation 1925/2006 to include new nutrient sources – specifically organic silicon (monomethylsilanetriol) and calcium phosphoryl oligosaccharides – in the authorized lists. It thus authorized these novel sources of silicon and calcium for use in supplements and fortified foods. This reflects the ongoing addition of EFSA‑approved micronutrient sources to EU law.
Delegated Regulation (EU) 2016/127 (71) (IF/FU Formula Composition)	2016	Infant formula composition	Supplements Regulation 609/2013 with detailed compositional rules for infant and follow-on formula. It specifies exact nutrient ranges – including precise vitamin and mineral content levels – to meet infants’ needs. This delegated act replaced Directive 2006/141/EC and updated formula standards (e.g. adding new nutrient sources) to ensure formula safety and adequacy.
Delegated Regulation (EU) 2016/128 (72) (FSMP Composition)	2016	Food for special medical purposes (FSMP)	Defines composition requirements for foods for special medical purposes (FSMP), including those for infants. It lists allowed vitamins, minerals and other nutrients under medical supervision, harmonizing previously divergent national rules (Directive 1999/21/EC was repealed). The Regulation ensures FSMP have specified nutrient profiles to manage medical conditions, though it may not cover all emerging ingredients.
Commission Regulation (EU) No 119/2014 (73)	2014	Fortified foods (chromium sources)	Amends Directive 2002/46 and Regulation 1925/2006 to authorize chromium-enriched yeast and chromium(III) lactate tri-hydrate as nutrient sources. Following positive EFSA opinions, it added these chromium forms to the EU lists. The regulation harmonizes EU rules with scientific advice but does not itself set fortification levels.
Regulation (EU) No 609/2013 (56) (Food for Specific Groups)	2013	Infant/young child foods and FSMP	Comprehensive rules for foods intended for infants, young children and for special medical purposes. Its Annex contains a single list of all vitamins and minerals that may be added to these foods, replacing multiple older directives. It sets general compositional and information requirements to protect these vulnerable groups. Specific nutrient content requirements are set by delegated acts, but this Regulation ensures only approved micronutrient sources are used.
Commission Regulation (EU) No 1161/2011 (74)	2011	Fortified foods (mineral sources)	Amends Directive 2002/46 and Regulation 1925/2006 to add new mineral substances to the authorized lists. It ensures that safe, EFSA‑approved new mineral nutrient sources become available for use in fortified foods and supplements. This act deals with permitted mineral forms; maximum usage levels are handled by separate EFSA and regulatory processes.
Commission Regulation (EC) No 1170/2009 (75)	2009	Fortified foods & supplements (vitamin/mineral sources)	Amends Directive 2002/46 and Regulation 1925/2006 to update EU lists of permitted vitamin and mineral substances and their forms. It replaced the Annexes of 2002/46/EC and expanded the range of approved micronutrient sources in foods and supplements. The Regulation’s focus is on authorizing new nutrient sources (e.g. new chemical forms), without setting usage level limits.
Regulation (EC) No 108/2008 (76)	2008	Fortified foods (general)	Amends Regulation (EC) No 1925/2006 introducing the regulatory procedure with scrutiny for adopting or amending measures related to the addition of vitamins, minerals, and certain other substances to foods. It empowers the Commission to set or update conditions such as maximum/minimum levels, purity criteria, and to manage the inclusion of substances in Annex III based on health risks. Enhances oversight while maintaining consumer safety and scientific assessment through EFSA.
Commission Directive 2006/141/EC (77) (Infant Formula)	2006	Infant formula	Sets compositional and labeling requirements for infant formula and follow-on formula. Its annexes specify required levels (minima/maxima) of energy, protein, fat, carbohydrate, vitamins and minerals. The Directive ensures formulas meet infants’ nutritional needs safely; it harmonized earlier national laws but only defines nutrient ranges rather than mandating additional fortification beyond those levels.
Commission Directive 2006/125/EC (78) (Processed Cereal-Based and Baby Foods)	2006	Baby (weaning) foods	Governs the composition of processed cereal-based and other baby foods used in weaning. It prescribes nutrient criteria (protein, fat, carbohydrates, vitamins and minerals) including minimum and maximum levels. This harmonizes nutrient content in complementary foods for infants/young children to ensure essential micronutrients are provided without exceeding safe limits.
Regulation (EC) No 1925/2006 (45) (Addition of Vitamins and Minerals)	2006	Fortified foods (general)	Harmonizes the addition of vitamins, minerals and certain other substances to *all* foods. It provides EU-wide positive lists (Annex I for vitamins/minerals, Annex II for sources) and requires EFSA safety assessments for new substances. The Regulation facilitates voluntary food fortification and enrichment while maintaining consumer protection, though it does not mandate any food to be fortified.
Directive 2001/15/EC (79)	2001	Foods for nutritional uses	Lists vitamins, minerals, amino acids, and other nutrients allowed in foods for specific uses. Enables flexibility in formulation across nutritional needs.
Directive 96/5/EC (80)	1996	Processed cereal-based and baby foods	Sets nutritional and safety criteria for infant and young child cereals. Permits voluntary fortification using specified substances to support nutritional adequacy.

A cornerstone of EU regulation in the area of food fortification is Regulation (EC) No 1925/2006 on the addition of vitamins and minerals to foods. This regulation provides EU-wide lists of permissible vitamins, minerals, and their chemical forms that may be added to food products. It establishes clear procedures for the evaluation and authorization of new nutrient sources, ensuring additions are scientifically justified and safe for consumers. Importantly, it creates a harmonized baseline for voluntary fortification and sets safety rules applicable to all fortification (voluntary or mandatory). Article 11 is the critical provision that explains the observed policy divergence. It deliberately allows EU Member States the flexibility to implement mandatory national fortification programs tailored to their specific needs, possibly leading to the patchwork of mandatory and voluntary approaches across Europe, described in the previous section, alongside the independent policies of non-EU states. The regulation enables diversity in mandatory action while harmonizing the underlying rules for safety and voluntary practices.

To ensure that legislative measures are evidence-based and scientifically sound, the EU extensively relies on the expertise of the European Food Safety Authority (EFSA). EFSA provides independent scientific advice on nutrition and health matters, evaluates the safety and bioavailability of nutrient sources, and advises on appropriate intake levels, thus forming the scientific backbone of EU nutritional regulation. EFSA’s assessments inform legislative updates, including revisions to permitted nutrient lists, new fortification practices, and maximum nutrient levels, helping to adapt European food law to the latest scientific insights and public health needs. EFSA’s evaluations are also a prerequisite for the European Commission to amend the list in Regulation (EC) No 1925/2006 (45). Only nutrient sources that receive a positive EFSA opinion regarding their safety and bioavailability can be considered for inclusion in Annexes I and II of the Regulation (55).

However, implementing food fortification policies and related food information regulations within the EU has previously and still faces several significant challenges. Achieving harmonization across Member States is particularly complex due to national variations and differing interpretations of EU regulations (56). Such differences can impede the free movement of fortified foods, create unequal competition, and generate legal uncertainty. An example of this complexity can be found in the 2004 European Court of Justice (ECJ) judgment in Commission v Netherlands (C-41/02) (57), which highlighted tensions between national fortification requirements and EU single-market principles. The Dutch requirement for a demonstrated “nutritional need” before allowing fortified foods onto the market was struck down by the Court as disproportionately restrictive and incompatible with EU law. As a result, the Netherlands revised its approach, introducing risk-based exemptions focused on upper intake limits. This case underscored the necessity of aligning national regulatory measures with substance-specific, scientific risk assessments to avoid barriers to trade, and it remains a key reference point in efforts to harmonize fortification policies across Member States.

Additionally, the dynamic nature of scientific progress and the broad range of products covered pose challenges to regulatory clarity and consistency. Definitions and exhaustive listings of permitted nutritional substances are challenging due to product diversity and evolving manufacturing processes. Monitoring compliance and enforcing regulations, especially regarding misleading information and promotional restrictions for vulnerable consumer groups like infants further adds to these complexities (56). The scientific substantiation of health claims and accurate dietary intake assessment also require rigorous methodologies, presenting a considerable burden of proof on both industry and regulatory bodies (58).

Overall, the EU’s legislative approach aims to balance public health protection with market harmonization, addressing diverse national contexts through flexible yet clearly defined regulatory frameworks, consistently informed by scientific assessments from EFSA.

To clarify how EU micronutrient legislation is developed and implemented, Table 3 outlines the key institutions involved, their roles, and associated legal instruments. Central to EU micronutrient policy development is the EFSA, which provides independent scientific assessments regarding nutrient safety, bioavailability, and recommended intake levels, as mentioned above. These scientific opinions serve as the evidence-based foundation for policy formulation. The European Commission, specifically its Directorate General for Health and Food Safety (DG SANTE), translates EFSA’s assessments into legislative proposals or amendments, typically adopted through delegated or implementing acts. For significant legislative changes, proposals undergo the ordinary legislative procedure, requiring co-decision by the European Parliament and the Council of the EU. National authorities are responsible for the domestic implementation, monitoring, and enforcement of these EU frameworks. Beyond EU institutions, the World Health Organization Regional Office for Europe (WHO Europe) complements this governance structure by providing Member States with technical cooperation, policy guidance, and regional action plans. WHO Europe’s efforts contribute to harmonized, equity-focused approaches to tackling micronutrient deficiencies.

**Table 3:** Key actors, roles and legal instruments for EU food policy.

Actor	Role	Legal instrument
EFSA	Provides independent scientific assessments on nutrient safety, bioavailability, intake levels; advises on amendments to positive lists.	EFSA opinions [established by Regulation (EC) No 178/2002 (81)]
European Commission - DG SANTE	Drafts legislative proposals, develops delegated and implementing acts, oversees compliance	Treaty on the Functioning of the European Union (TFEU) (82); Delegated/Implementing acts under Regulation (EC) No 1925/2006 (45)
European parliament and council	Co-legislators for major changes to regulations and directives, adopt legislation through ordinary legislative procedure	TFEU Articles 114 (83) and 168 (59)
Member States	Implement or transpose regulations and directives, can establish national mandatory fortification programs within EU frameworks	Regulation (directly applicable) or Directive (requires national law through transposition)
National Food Safety Authorities	Enforce and monitor compliance	National legislation and monitoring programs
WHO Europe/Other International Partners	Provide technical guidance, facilitate capacity building and knowledge sharing	WHO Action Frameworks, joint declarations

Advancing policy change across Europe requires leveraging multiple pathways within this governance framework. At the EU level, mechanisms such as delegated or implementing acts under Regulation (EC) No 1925/2006 could refine fortification standards, while coordinated initiatives under Article 168 of the Treaty on the Functioning of the European Union (TFEU) (59) empower the EU to supplement and align Member State public health measures. The European Council can further support harmonization through joint recommendations. Additional policy instruments, such as conditioning EU funding or procurement rules on alignment with evidence-based fortification standards, or incentivizing industry reformulation through public-private partnerships, could enable more responsive and effective fortification strategies across Member States. While such measures may support transitions from voluntary to mandatory schemes where appropriate - guided by national needs, equity considerations, and scientific evidence -, their primary aim is to foster coherent, context-sensitive approaches that improve micronutrient intake and public health outcomes. Together with participatory stakeholder engagement and evidence-informed advocacy, these coordinated approaches hold significant potential to accelerate equitable and sustainable improvements in micronutrient status across Europe.

### 3.3 Complementary policy instruments addressing micronutrient deficiencies

#### 3.3.1 Nutrition labeling, health claims, and nutrient profiling

Under EU regulation No 1169/2011, nutrition labeling is mandatory for most pre-packed foods but front-of-pack (FOP) schemes remain voluntary. Regarding vitamin and mineral content, it can be added as extra nutritional details beyond the mandatory one. However, voluntary information can only be added provided they do not compromise the visibility or space reserved for mandatory information, which includes the energy value and amounts of fat, saturates, carbohydrate, sugars, protein, and salt (60). Additionally, complementary to Regulation 1169/2011, Regulation (EC) No 1924/2006 establishes the framework for nutrition and health claims, ensuring that any statements regarding micronutrient content (e.g., “source of iron”) are scientifically substantiated and appear only when relevant thresholds are met.

Across the EU market, several public-sector-supported FOP schemes are currently in use, reflecting a diverse regulatory landscape. Among these, the Keyhole logo is used in Sweden, Denmark, and Lithuania, while the Nutri-Score system has been adopted in France and Belgium, with plans for future implementation in Germany, Spain, the Netherlands, and Luxembourg. Other active schemes, often focused on cardiovascular health, include the Finnish Heart Symbol, Slovenia’s ‘Little Heart’ logo, Croatia’s ‘Healthy Living’ label, and the Traffic Light system used in Ireland. Italy has developed its own scheme, the NutrInform Battery, which has received official backing but has not yet been rolled out (60). Although these schemes aim to support healthier food choices by simplifying nutritional information, they vary in scope and methodology, and importantly none of them account for the micronutrient density in their calculations.

While FOP labeling schemes contribute to clearer food information, their reliance on macronutrient-based scoring systems and omission of micronutrients has been widely criticized. This omission may limit their effectiveness in promoting nutritionally adequate diets, especially for at-risk groups. Academic analyses, particularly of Nutri-Score, have called for future adaptations that integrate micronutrient density to better align front-of-pack labels with broader public health nutrition goals (61).

#### 3.3.2 Food insecurity and social policy responses

Micronutrient intake is closely tied to food access and affordability. Policies that address food insecurity, such as food subsidies, social protection alignment, and emergency aid, can have significant indirect effects on micronutrient status.

One of the flagship EU-level instruments has been the Fund of the European Aid to the Most Deprived (FEAD), which provided food assistance and basic goods to vulnerable populations (62). Since 2021, FEAD activities have been integrated into the European Social Fund Plus (ESF+), which offers greater flexibility in delivery mechanisms, allowing the use of e-vouchers. These schemes are designed to reduce logistical costs, enhance efficiency, offers beneficiaries more autonomy and dignity, and reduces stigma associated with receiving food aid (63). Several countries adopted the scheme, including Belgium, France, Italy, Lithuania, and Spain.

At the national level, several countries have pioneered dignity-based, nutrition-sensitive food assistance models. In Estonia, the Ministry of Social Affairs replaced the standardized food parcel system, previously reliant on fixed packages of non-perishable items distributed through local agencies, with a nationwide Foodcard program in 2023. This shift allows low-income households to independently purchase food from regular retailers, leading to increased purchases of fresh produce, dairy, meat, and fish. The program also enables precise tracking of food purchasing trends and demonstrates a shift toward healthy eating choices, while significantly reducing logistical barriers and social stigma (64). Similarly, in France, the ANDES solidarity grocery store network offers a scalable model that combines food aid with community engagement and nutritional support. These stores allow eligible individuals to buy subsidized groceries, including fresh produce, dairy, and other nutrient-rich foods, while also accessing workshops, personalized support, and social inclusion activities. According to ESF+, ANDES plans to expand its reach by opening new stores and launching mobile units to serve underserved rural areas (65).

Though not including explicit evaluation frameworks for micronutrient outcomes, both cases illustrate a trend toward empowering beneficiaries with choice, improving nutritional quality of assistance, and reducing access barriers.

## 4 Discussion

Although Europe overcame the most severe forms of undernutrition during the post-World War II recovery, persistent micronutrient deficiencies remain a significant public health challenge across the continent. To our knowledge, this is the first comprehensive policy review of micronutrient fortification in Europe, addressing a critical knowledge gap not explored in depth in previous global or sector-specific reviews. By analyzing policy approaches and opportunities for harmonization, this study complements broader evaluations of fortification programs worldwide, providing a detailed and policy-focused perspective on addressing micronutrient deficiencies through food within the European context.

This work investigated the demographic, socioeconomic, and geographical determinants of micronutrient deficiencies across Europe and searched for and analyzed the national and regional policy responses. Hidden hunger remains a significant, inequitably distributed public health burden, disproportionately affecting vulnerable groups due to intersecting biological needs, economic constraints, dietary patterns – increasingly favoring processed and nutrient-poor foods –, and environmental factors.

While large-scale food fortification cannot replace a diverse, nutrient-rich diet and may have limited impact on populations outside formal food markets, it remains a critical public health intervention. When effectively combined with supplementation and targeted nutritional strategies, fortification significantly contributes to advancing nutrition security and health equity. While many countries have implemented comprehensive, mandatory strategies for some micronutrients, many others rely on limited or voluntary approaches, and targeting only few of the needed micronutrients, resulting in uneven population coverage and persistent deficiencies. The findings reveal significant fragmentation in these policies, with notable differences in the scope and effectiveness of fortification efforts. Additionally, the review identifies challenges in the harmonization of regulations among EU Member States, underlining the need for more coordinated and consistent approaches to tackle micronutrient deficiencies across the region. Salt iodization is the most widespread and historically successful mandatory fortification policy. However, implementation is highly fragmented: only 7 of the 33 countries analyzed in this review mandate universal iodization, while 6 have partial mandates (e.g., bakery salt, school meals), and 20 rely on voluntary schemes or lack policies. Beyond iodine, national fortification policies may also target vitamin D, A, folic acid, iron, and calcium. This inconsistency highlights the missed public health potential of coordinated evidence-based fortification policies at the European level and stresses the crucial role of robust national and coordinated pan-European regulatory frameworks.

Significant barriers impede the effective impact of current policies. The reliance solely on fortification, without broader supportive policies such as the implementation of micronutrient-inclusive nutrition labeling systems (e.g., Nutri-Score, Keyhole), subsidies for nutrient-rich foods or more comprehensive social protection measures, limits the ability to effectively address underlying issues like affordability and access. Moreover, regulatory fragmentation, characterized by disparate national approaches (mandatory vs. voluntary, differing target foods and nutrient levels), hinders harmonized implementation, creates trade barriers, and limits equitable coverage. Implementation gaps further exacerbate these issues, as mandatory frameworks often suffer from weak monitoring and enforcement, and voluntary schemes experience variable industry compliance and consumer awareness. In this context, alignment between public health objectives and private sector practices is essential. Stronger public–private coordination, supported by clear regulatory guidance and monitoring mechanisms would help ensure that food policies deliver consistent public health benefits rather than uneven, market-driven outcomes (66). Ultimately, policy silos prevent cohesive integration between strategies and approaches.

Policymakers should reinforce mandatory fortification strategies informed by robust scientific evidence, focusing on prevalent deficiencies. Simultaneously, improving access to nutritious foods through targeted economic incentives and subsidies will address underlying socioeconomic constraints. Enhancing regulatory compliance through strengthened monitoring systems, rigorous enforcement, and robust data collection infrastructures is crucial. Additionally, comprehensive public education initiatives should be developed to improve nutrition literacy and foster sustainable dietary improvements across all populations.

### 4.1 Theory of change to improved micronutrients status

To effectively confront these challenges, a shift toward coordinated, mandatory, and equity-sensitive fortification embedded within comprehensive food and social policies is required. To effectively translate regulatory actions into tangible public health improvements, this review proposes a theory of change that systematically maps the causal pathway linking regulatory inputs at the EU level to improved individual micronutrient status, explicitly highlighting essential enabling conditions and feedback mechanisms. Initially, essential inputs are crucial to policy formulation. These include harmonized regulatory frameworks, robust scientific evidence, sufficient funding and resources, and meaningful stakeholder engagement – marginalized populations *in primis* – for the co-development of effective and equitable recommendations and regular evaluation of the effectiveness of policy changes. These inputs lead to policy outputs, including harmonized legislation, mandatory fortification schemes, clear labeling requirements, and targeted supplementation programs. Successful national implementation involves effective transposition of EU regulations, reliable enforcement mechanisms, consistent compliance monitoring, and active citizen participation. Consequently, these implementation outcomes enhance intermediate outcomes, notably increasing the availability and accessibility of fortified foods, improving dietary diversity, and heightening nutrition literacy and consumer awareness. Equity-sensitive mechanisms are embedded across stages of this pathway, with metrics the include differential coverage, affordability, and utilization among priority populations and settings, to inform course-corrections through monitoring and evaluation. Ultimately, this structured approach results in significant public health impacts, reducing micronutrient deficiencies, enhancing physical and cognitive development, decreasing morbidity and mortality, and promoting greater health equity across Europe.

A systematic consideration of stakeholder roles is essential for translating policy inputs into public health outcomes. Across Europe, stakeholder and consumer engagement in the process of setting micronutrient recommendations is uneven, with wide cross-country variation in the involvement of government, civil society, and industry actors (67). This underscores the need to incorporate diverse voices into nutrition policy and to reconcile EU-level harmonization with sensitivity to national and local contexts. National governments establish regulatory frameworks and monitoring systems that set the scope and stringency of policy instruments. Local and regional authorities operationalize policies in communities and institutions, for example through public procurement food standards. Civil society and consumer organizations contribute advocacy, inclusion, and accountability, helping align strategies with population needs and equity goals. Academic and scientific institutions generate and appraise evidence and conduct risk assessment underpinning EU legislation and national guidance. Health systems and professional bodies deliver supplementation, counseling, and case-finding. International organizations (WHO, FAO, European Commission) provide harmonization, technical assistance, and surveillance frameworks. Finally, industry actors shape availability, formulation, labeling, and distribution of nutritious food; their engagement and compliance affect coverage and effectiveness across the full policy spectrum.

### 4.2 Points of concern and areas for policy innovation

While this review highlights the significant progress made in tackling micronutrient deficiencies across Europe, it also reveals persistent gaps and emerging challenges that merit further attention.

(1) There is a need to move beyond a narrow micronutrient focus toward a more holistic, diet-quality-based approach. Micronutrient deficiencies often coexist with excessive intakes of unhealthy macronutrients (e.g., saturated fats, sugars, and sodium) and energy-dense, nutritionally-poor foods. A more integrated policy framework, considering both macro- and micronutrient quality, would better address the double burden of malnutrition and diet-related NCDs. (2) Despite the existence of EU-level harmonized frameworks, fortification policies and practices remain highly fragmented across Member States, creating inconsistencies and unequal protection of vulnerable and marginalized groups. Future policy recommendations should prioritize equity-sensitive, harmonized, and evidence-based fortification standards, while still respecting national and cultural contexts. (3) Current monitoring and evaluation systems for fortification policies are often insufficient or not sustained over time, exception for a few well-documented cases such as Finland. More robust, transparent, and mandatory monitoring frameworks could ensure accountability, track long-term health outcomes, and identify unintended consequences early. (4) Reliance on voluntary or recommended industry action leaves room for inconsistent implementation. Strengthening accountability mechanisms, including clearer incentives for industry compliance or mandatory minimum fortification requirements for high-risk nutrients, would help close this gap. (5) Policies should explicitly address equity as a guiding principle, ensuring that fortification and supplementation strategies reach those most affected by social, economic, and geographic vulnerabilities. A participatory policy development process, involving civil society, vulnerable groups, and independent scientific experts, would strengthen legitimacy and effectiveness.

These considerations highlight the importance of embedding any future European fortification strategy within a broader, equity-driven, diet-quality-based policy framework. This approach will be essential to ensure sustainable, effective, and socially just solutions to hidden hunger across Europe.

## 5 Conclusion

Despite improvements in food availability, healthcare, and general living standards, micronutrient deficiencies remain a persistent challenge across Europe. This review highlights biological, social, and structural determinants that drive inequities in micronutrient intake, with disproportionately high burdens among children, females of reproductive age, older adults, low-income populations, and those living in low-UV regions. Hidden hunger, defined by insufficient intake of essential micronutrients despite adequate caloric consumption, continues to undermine health, productivity, and wellbeing throughout life course, often without overt clinical indicators. These deficiencies contribute not only to short- and long-term morbidity but also to intergenerational cycles of disadvantage.

At the policy level, although several countries have introduced targeted food fortification policies, these remain fragmented in scope and variable in implementation. Most fortification strategies across Europe are still voluntary, leading to inconsistent coverage and limited impact. While EU legislation [particularly Regulation EC No 1925/2006 (45)] provides a harmonized framework for addition of micronutrients to foods, flexibility has allowed wide divergence in national approaches, reducing opportunities for coherent, large-scale public health gains.

Nonetheless, evidence indicates that well-designed fortification policies - especially when mandatory, regulated, and monitored - can significantly improve micronutrient status at the population level. The example of Finland’s vitamin D strategy illustrates how policy informed by public health surveillance can drive measurable improvements. Other countries have shown that targeted fortification, particularly of staple foods, can be effective and cost-efficient. What remains lacking is a coordinated European response that fully integrates equity into policy design and implementation. Addressing hidden hunger demands more than technical solutions; it requires political will, intersectoral collaboration, and a commitment to reducing health inequities. Without specific attention to the structural and social determinants of hidden hunger, fortification alone will not be sufficient to overcome existing disparities.

The Horizon-funded Zero Hidden Hunger EU project represents a timely and important step toward bridging this policy gap. By fostering collaboration among researchers, policymakers, and civil society actors, it offers a model for participatory policy co-creation grounded in scientific evidence and lived experience. It also highlights the need to move beyond technical interventions toward systemic solutions that are sensitive to the diverse realities of European populations. Tools and knowledge to eliminate micronutrient deficiencies in Europe already exist. What is required now is political leadership, regulatory alignment, and strategic investment to ensure that every individual - regardless of age, sex, income, or location - can meet their basic nutritional needs. Tackling hidden hunger is not only a matter of health promotion but a moral imperative: a prerequisite for achieving social justice, economic resilience, and the broader vision of a sustainable, inclusive Europe.
