# Supplementary material for: Hidden hunger in Europe: a review on determinants, fragmented policy responses, and implementation barriers
Source: Front Nutr. 2025 Oct 20;12:1669008. doi: 10.3389/fnut.2025.1669008 (PMC12580594; doi:10.3389/fnut.2025.1669008)
Supplement: Supplementary file 1 [file Table_1.docx]

**Supplementary Table 1 and Sources: National salt iodization legislation in the European Economic Area region**. Adapted from *Prevention and control of iodine deficiency in the WHO European Region adapting to changes in diet and lifestyle, 2024* (1)

| **Country** | **Legislation name & year** | **Legal status** | **Permitted iodine source** | **Iodine content (mg/kg salt)** |
| --- | --- | --- | --- | --- |
| **Austria** | National Council of the Republic of Austria. Federal Law on the trade of table salt. 1963 (2) (updated 1990 (3) and 1999 (4)) | Mandatory for the production of bread and bakery products intended for sale | KI/KIO₃ | 1963–1990: 7.5 mg I |
|  |  |  |  | 1990–1999: 15 mg I |
|  |  |  |  | 1999–present: 15–20 mg I |
| **Belgium** | No legislation. Voluntary agreement on iodized salt in bread between the Belgian bakery sector and the Federal Ministry of Health. 2009 (5) | Voluntary | KI/KIO_3_/NaI | 15 mg I (recommendation) |
| **Bulgaria** | Council of Ministries. Decree No. 96 of the Council of Ministers on determining measures for the prevention and eradication of iodine deficiency diseases and disorders (introduces universal salt iodization using KIO₃). 1994 (6) (repealed) | Mandatory for all salt for human consumption | KIO₃ | 1994–2001: No specified levels |
|  | Council of Ministries. Decree No. 23 of the Council of Ministers on requirements for the composition and characteristics of food-grade salt. 2001 (7) |  |  | 2001–present: 28–55 mg KIO₃/ 17–33 mg I/ |
| **Croatia** | Salt iodination law in Yugoslavia. 1953 (8) | Mandatory for all salt for human consumption | KI/KIO₃/NaI/NaIO_3_ | 1953–1996: 10 mg KI/7.5 mg I |
|  | Instruction on the iodization of table salt (Official Gazette 84/96). 1996 (9) |  |  | 1996–2010: 25 mg KI/19.1 mg I |
|  | Ministry of Agriculture, Fisheries and Rural Development. Ordinance on salt (94/2010). 2010 (10) |  |  | 2010–present: 20–30 mg KI/15–23 mg I |
|  | Ministry of Agriculture. Ordinance on salt (70/2019). 2019 (11) |  |  |  |
| **Cyprus** | No fortification (1) | | | |
| **Czech Republic** | Ministry of Agriculture. Decree No. 331 on food and tobacco products and on amendments and supplements to certain related acts concerning spices, edible salt, dehydrated products, flavorings, and mustard. 1997 (12) (repealed) | Voluntary | KI/KIO_3_/NaI/NaIO_3_ | 1997–present: 20–34 mg I |
|  | Ministry of Agriculture and Food. Decree No. 398 on requirements for spices, edible salt, dehydrated products, flavorings, cold sauces, dressings and mustard. 2017 (13) |  |  |  |
| **Denmark** | Food Directorate of Denmark. Order 627 on the addition of iodine to household salt and salt in bread and general bakery products etc. 2000 (14) (repealed 2014) | Mandatory for household salt and for the production of bread and bakery products intended for sale | KI/KIO_3_/NaI/NaIO_3_ | 2000–2019: 13 mg I |
|  | Ministry of Environment and Food. Order 613 on the addition of iodine to household salt and salt in bread and general bakery products etc. 2014 (15)(repealed 2019) |  |  |  |
|  | Ministry of Environment and Food. Order 1318 on the addition of iodine to household salt and salt in bread and general bakery products etc. 2019 (16) |  |  | 2019–present: 20 mg I |
| **Estonia** | No fortification (1) | | | |
| **Finland** | National Nutrition Council. General principles and requirements of food law establishing the European Food Safety Authority and laying down procedures in matters of food safety. 2002 (17) | Voluntary | – | 25 mg I (recommendation) |
| **France** | Ministry of Economy, Finance and Industry, Ministry of Agriculture and Fisheries and Ministry of Health and Solidarity. Order on nutritional intake substances that may be used for the supplementation of salt for human consumption. 2007 (18) | Voluntary | KI/KIO_3_/NaI/NaIO_3_ | 15–20 mg I |
| **Germany** | Federal Minister of Youth, Family, Women and Health. Regulation amending the requirements for iodized table salt. 1989 (19) | Voluntary Regulation amending the requirements for iodized table salt | KIO₃/NaIO₃ | 15–25 mg I |
|  | Bundesrat Federal Council. Second regulation amending the provisions on iodized table salt. Germany. 1993 (20) |  |  |  |
| **Greece** | Ministry of Rural Development and Food. Ministerial Decree No. 1100/1987 codifying the provisions of the Food Code. 1987 (21) | Voluntary | KI | 40–60 mg I |
| **Hungary** | Ministry of Human Resources. Decree 37/2014 on nutrition and health regulations in mass catering. 2014 (22) (replaces MSZ-01-10007:1982 (23)) | Mandatory for public catering food preparation (education, health, and social welfare facilities) | KI/KIO₃ | ≤ 25 KI, ≤ 32.2 KIO_3_ |
| **Iceland** | No fortification, national policy or recommendation (1,24) | | | |
| **Ireland** | No fortification (1) | | | |
| **Italy** | Ministry of Health. Decree No. 562 Regulation concerning the production and commercialization of table salt and iodized salt. 1995 (25) | Voluntary (authorized for production and sale; promoted for iodine deficiency prevention but not mandatory) | KI/KIO₃ | 24–42 mg I |
|  | President of the Republic. Law 21 March 2005 No. 55 Provisions aimed at the prevention of endemic goiter and of other iodine deficiency diseases. 2005 (26) | Mandatory for specific distribution channels: shops must sell both iodized and non-iodized salt but are required to only provide non-iodized salt on specific requests. It also requires the use of iodized salt in restaurants and allows production of processed foods with iodized salt. |  |  |
| **Latvia** | Cabinet Regulation No. 488 on minimum safety, quality, hygiene and labelling requirements for edible salt and requirements for the distribution and use of salt in food production. 2005 (27) (repealed) | Voluntary | KI/KIO₃/NaI/NaIO₃ | Table or common salt: 0.002–0.005% iodine or 20–50 mg I  Salt for food production: 0.004–0.01% or 40–100 mg I |
|  | Cabinet Regulation No. 696 regarding edible salt. 2015 (28) |  |  |  |
| **Liechtenstein** | Federal Department of Home Affairs (FDHA) Ordinance on the addition of vitamins, minerals and other substances in foodstuffs. 2005 (29) (repealed) | Voluntary | KI/KIO₃/NaI/NaIO₃ | 2006–2017: 20–30 mg I |
|  | Federal Department of Home Affairs (FDHA) Ordinance on the addition of vitamins, minerals and other substances in foodstuffs. 2016 (30) (updated salt iodization values) |  |  | 2017–present: 20–40 mg I |
| **Lithuania** | Minister of Health. Amendment of the Lithuanian Hygiene Standard HN 15:2005. 2005 (31) | Mandatory for all table salt sold in retail stores and salt used in public catering/bakeries | Unspecified | 20–40 mg I |
| **Luxembourg** | No fortification (1) | | | |
| **Netherlands (the Kingdom of)** | Commodities Act Decree on the addition of micronutrients to food. 1999 (32) (repealed) | Voluntary | KI/KIO₃/NaI/NaIO₃ | 1999–2008: salt 30–40 mg I  bread and cereal-based products 70–85 mg I  meat and meat products 20–30 mg I |
|  | Commodities Act Decree on the addition of micronutrients to food. 2008 (33) (repealed) |  |  | 2008–present: bread, bread substitutes, other bakery products max 65 mg I, other foodstuffs max 25 mg I |
|  | Commodities Act Decree on the addition of micronutrients to food. 2014 (34) |  |  |  |
| **Norway** | Ministry of Health and Welfare. Regulation on the addition of vitamins, minerals and certain other substances to foodstuffs. 2010 (35) | Voluntary | Unspecified | Max 20 mg I |
| **Poland** | Minister of Health Regulation on enriching substances added to food and conditions of their use. 2002 (36) (repealed) | Mandatory for table salt | KI/KIO₃ | 15.3–30.7 mg I/ 20–40 mg KI/ 26–52 mg KIO₃ |
|  | Minister of Health. Regulation on enriching substances added to food and conditions of their use. 2010 (37) |  |  |  |
|  | Minister of Health. Regulation on enriching substances added to food and conditions of their use and prohibited substances. 2024 (38) |  |  |  |
| **Portugal** | Ministry of Health. Decree-Law no. 87/96, Official Gazette Series I-A, no. 152. 1986 (39) | Voluntary for retail salt | KI | 25–35 mg KI/ 19–26.6 mg I |
|  | Ministry of Education. Circular no. 3/DSEEAS/DGE/2013 School guidelines. 2013 (40) | Mandatory for school meals since 2013 |  |  |
|  | Ministry of Education. Guidelines on school menus and canteens (update). 2018 (41) |  |  |  |
| **Romania** | Decision no. 568/2002 on the universal iodization of salt for human consumption, animal feed and use in the food industry. 2002 (42) (amended by Decision no. 473/2004 (43) & 1.904/2006 (44)) | Mandatory for all salt for human consumption | KI/KIO₃ | 25–40 mg I/ 32.5–52 mg KI/ 42–67.2 mg KIO₃ |
| **Slovakia** | Ministry of Agriculture and Rural Development. Decree 309/2015 on snacks, edible salt, dehydrated dishes, soups and flavourings. 2015 (45) | Mandatory for all salt for human consumption | KI/KIO₃ | 15–35 mg KI or KIO₃/ 11.5–26.8 mg I |
| **Slovenia** | Salt Iodination Law in Yugoslavia. 1953 (8,46) | Mandatory for all salt for human consumption (excluding sea salt since 2013) | KI/KIO₃ | 1953–1999: 10 mg KI/7.5 mg I |
|  | Minister of Health. 2659 Rules on tea, mustard, table salt, baking powder, pudding powder 1999 (47) |  |  | 1999–2003: 20–30 mg KI/26–39 mg I |
|  | Minister of Agriculture, Forestry and Food. 3397 Rules on the quality of salt. Official Gazette of the Republic of Slovenia. 2003 (48) |  |  | 2003–present: 20–30 mg KI/26–39 mg KIO₃/16–23 mg I |
| **Spain** | Presidency of the Government. Royal Decree 1424/1983, of April 27, approving the technical sanitary regulation for obtaining, circulating and selling of salt and edible brines. Spain. 1983 (49) | Voluntary | KI/KIO₃ | 51–69 mg I |
|  | Ministry of Consumption. Protocol of minimum criteria for the evaluation of food supply in schools: school canteens, food and beverage vending machines and cafeterias in accordance with program 16 of the national plan for the official control of the food chain 2021–2025. 2022 (50) |  |  |  |
| **Sweden** | Government of Sweden. Regulations on the fortification of certain foods: SLVFS 1983:2 (51) (amended by LIVSFS 2002:19 (51)) Regulations on enrichment of certain foods. 2002 | Voluntary | Not mentioned | 1983–2007: 40–70 mg I |
|  | LIVSFS 2007:9 (52) – Follows EU Regulation (EC) No 1925/2006 on the addition of vitamins and minerals and of certain other substances to foods. It allows iodine and fluoride to be added to foods and indicates allowed compounds but does not establish standards or mandatory fortification. |  |  | 2007–present: no established standards (probably 40–70 mg I) |
| **Switzerland** | Federal Department of Home Affairs (FDHA) Ordinance on the addition of vitamins, minerals and other substances in foodstuffs. 2005 (29) (repealed) | Voluntary | KI/KIO₃/NaI/NaIO₃ | 2006–2017: 20–30 mg I |
|  | Federal Department of Home Affairs (FDHA) Ordinance on the addition of vitamins, minerals and other substances in foodstuffs. 2016 (30) (updated salt iodization values) |  |  | 2017–present: 20–40 mg I |
| **Türkiye** | Ministry of Food, Agriculture and Livestock. Turkish Food Codex Salt Communique No. 2007/53. 2008 (53) (allowed voluntary fortification of food industry salt and required iodization of all table salt) | Mandatory for all salt for human consumption | KIO₃ | 24–40 mg KIO₃ |
|  | Food, Agriculture and Livestock Minister. Turkish Food Codex Salt Communique. 2013 (54) |  |  |  |
| **Ukraine** | State Consumer Standard of Ukraine. Iodized Salt – Technical Specifications (DSTU 4307:2004). 2004 (55) | Voluntary | KI/KIO₃ | 25–55 mg I |
| **United Kingdom** | No fortification, national policy or recommendation (1,56) | | | |

# Sources

1. World Health Organization RO for Europe. Prevention and control of iodine deficiency in the WHO European Region adapting to changes in diet and lifestyle [Internet]. 2024 [cited 2025 Jul 17]. Available from: https://www.who.int/europe/publications/i/item/9789289061193

2. Austria. Federal Law of 17 April 1963 on the trade of table salt [Bundesgesetz vom 17 April 1963 über den Verkehr mit Speisesalz]. National Council [Internet]. 1963 [cited 2025 Jul 17]. Available from: https://www.ris.bka.gv.at/Dokumente/BgblPdf/1963_112_0/1963_112_0.pdf

3. Austria. Federal Law of 16 May 1990 amending the Federal Law on the trade in table salt [Bundesgesetz vom 16. Mai 1990, mit dem  das Bundesgesetz über den Verkehr mit  Speisesalz geändert wird]. National Council [Internet]. 1990 [cited 2025 Jul 17]. Available from: https://www.ris.bka.gv.at/Dokumente/BgblPdf/1990_288_0/1990_288_0.pdf

4. Austria. Federal Law of 20 July 1999 amending the Federal Law on the trade in table salt [Bundesgesetz vom 22. Juli 1999, mit dem  das Bundesgesetz über den Verkehr mit  Speisesalz geändert wird]. National Council of the Republic of Austria [Internet]. 1999 [cited 2025 Jul 17]. Available from: https://www.ris.bka.gv.at/Dokumente/BgblPdf/1999_115_1/1999_115_1.pdf

5. Vandevijvere S. Sodium reduction and the correction of iodine intake in Belgium: Policy options. Archives of Public Health [Internet]. 2012 May 30 [cited 2025 Jul 17];70(1). Available from: https://archpublichealth.biomedcentral.com/articles/10.1186/0778-7367-70-10

6. Bulgaria. Decree No. 96 of the Council of Ministers of 17 May 1994 on determining measures for the prevention and elimination of iodine deficiency diseases and disorders [ПОСТАНОВЛЕНИЕ № 96 НА МИНИСТЕРСКИЯ СЪВЕТ ОТ 17 МАЙ 1994 Г. ЗА ОПРЕДЕЛЯНЕ НА МЕРКИ ЗА ПРЕДОТВРАТЯВАНЕ И ЛИКВИДИРАНЕ НА ЙОДДЕФИЦИТНИТЕ ЗАБОЛЯВАНИЯ И НАРУШЕНИЯ]. Council of Ministries [Internet]. 1994 [cited 2025 Jul 17]. Available from: https://lex.bg/index.php/bg/laws/ldoc/-1087875069

7. Bulgaria. Decree No. 23 of 30.01.2001 on requirements for the composition and characteristics of food-grade salt [НАРЕДБА ЗА ИЗИСКВАНИЯТА КЪМ СЪСТАВА И ХАРАКТЕРИСТИКИТЕ НА СОЛТА ЗА ХРАНИТЕЛНИ ЦЕЛИ]. Council of Ministries [Internet]. 2001 [cited 2025 Jul 17]. Available from: https://lex.bg/bg/laws_stoyan/ldoc/-549185536

8. Vučinić M, Kušec V, Dundović S, Ille J, Dumić M. The effect of 17 years of increased salt iodization on the prevalence and nature of goiter in Croatian schoolchildren. Journal of Pediatric Endocrinology and Metabolism [Internet]. 2018 Sep 1 [cited 2025 Jul 17];31(9):995–1000. Available from: https://www.degruyterbrill.com/document/doi/10.1515/jpem-2018-0129/html

9. Mišljenje Z. Croatian Food Agency scientific opinion on the possibility of iodising desert salt. [HRVATSKA AGENCIJA ZA HRANU ZNANSTVENO MIŠLJENJE O MOGUĆNOSTI JODIRANJA PUSTINJSKE SOLI]. 2016 Jun.

10. Croatia. Ordinance on salt (94/2010) [Pravilnik o soli]. Ministry of Agriculture, Fisheries and Rural Development [Internet]. 2010 [cited 2025 Jul 17]. Available from: https://narodne-novine.nn.hr/clanci/sluzbeni/2010_07_94_2627.html

11. Croatia. Ordinance on salt (70/2019) [Pravilnik o soli]. Ministry of Agriculture [Internet]. 2019 [cited 2025 Jul 18]. Available from: https://narodne-novine.nn.hr/clanci/sluzbeni/2019_07_70_1472.html

12. Czechia. Decree No. 331 on food and tobacco products and on amending and supplementing certain related acts, for spices, edible salt, dehydrated products and flavourings and mustard [Vyhláška Ministerstva zemědělství, kterou se provádí  §18 písm. a), d), h), i), j) a k) zákona č. 110/1997 Sb., o potravinách a tabákových výrobcích a o změně a doplnění některých souvisejících zákonů, pro koření, jedlou sůl, dehydratované výrobky a ochucovadla a hořčici]. Ministry of Agriculture [Internet]. 1997 [cited 2025 Jul 17]. Available from: https://www.zakonyprolidi.cz/cs/1997-331

13. Czechia. Decree No. 398 on requirements for spices, edible salt, dehydrated products, flavourings, cold sauces, dressings and mustard [Vyhláška č. 398/2016 Sb.]. Ministry of Agriculture and Food [Internet]. 2017 [cited 2025 Jul 17]. Available from: https://www.zakonyprolidi.cz/cs/2016-398

14. Denmark. Order 627 on the addition of iodine to table salt and salt in bread and general baked goods, etc. [Bekendtgørelse om tilsætning af jod til husholdningssalt og salt i brød og almindeligt bagværk m.v.]. Food Directorate of Denmark [Internet]. 2000 [cited 2025 Jul 17]. Available from: https://www.retsinformation.dk/eli/lta/2000/627

15. Denmark. Order 613 on the addition of iodine to household salt and salt in bread and general bakery products etc. [Bekendtgørelse 613 om tilsætning af jod til  husholdningssalt og salt i brød og almindeligt  bagværk m.v.]. Ministry of Environment and Food [Internet]. 2019 [cited 2025 Jul 17]. Available from: https://www.retsinformation.dk/eli/lta/2019/613

16. Denmark. Order 1318 on the addition of iodine to household salt and salt in bread and general bakery products etc. [Bekendtgørelse 1318 om tilsætning af jod til  husholdningssalt og salt i brød og almindeligt bagværk m.v.]. Ministry of Environment and Food [Internet]. 2014 [cited 2025 Jul 17]. Available from: https://www.retsinformation.dk/eli/lta/2014/1318

17. Finland. The National Nutrition Council recommendations. National Nutrition Council of Finland [Internet]. 2002 [cited 2025 Jul 17]. Available from: https://www.ruokavirasto.fi/globalassets/teemat/terveytta-edistava-ruokavalio/ravitsemus--ja-ruokasuositukset/eng/vrn_jodi_toimenpidesuositus_10_2.2015_english.pdf

18. France. Order on nutritional intake substances that may be used for the supplementation of salt for human consumption [Arrêté du 24 avril 2007 relatif aux substances d’apport nutritionnel pouvant être utilisées pour la supplémentation des sels destinés à l’alimentation humaine]. Ministry of Economy, Finance and Industry, Ministry of Agriculture and Fisheries and Ministry of Health and Solidarity [Internet]. 2007 [cited 2025 Jul 17]. Available from: https://www.legifrance.gouv.fr/loda/id/JORFTEXT000000645808/

19. Germany. Regulation amending the requirements for iodised table salt [Verordnung zur Änderung der Vorschriften über jodiertes Speisesalz]. Federal Minister of Youth, Family, Women and Health [Internet]. Regulation amending the requirements for iodized table salt [Verordnung zur Änderung der Vorschriften über jodiertes Speisesalz]. Federal Minister of Youth, Family, Women and Health. 1989 [cited 2025 Jul 18]. Available from: https://dejure.org/BGBl/1989/BGBl._I_S._1123

20. Germany. Second regulation amending the provisions on iodized table salt [Zweite Verordnung zur Änderung der Vorschriften  über jodiertes Speisesalz]. Bundesrat Federal Council [Internet]. 1993 [cited 2025 Jul 18]. Available from: https://dserver.bundestag.de/brd/1993/D554+93.pdf

21. Greece. Ministerial Decree No 1100/1987 codifying the provisions of the Food Code. Ministry of Rural  Development and Food [Internet]. 1987 [cited 2025 Jul 16]. Available from: https://www.fao.org/faolex/results/details/en/c/LEX-FAOC106642/

22. Hungary. Decree 37/2014 on nutrition and health regulations in mass catering. [EMMI rendelet 37/2014 a közétkeztetésre vonatkozó táplálkozás-egészségügyi előírásokról]. Ministry of Human Resources [Internet]. 2014 [cited 2025 Jul 17]. Available from: https://njt.hu/jogszabaly/2014-37-20-5H

23. Hungary. Standard MSZ11007 for food grade salt (sodium chloride) [MAGYAR SZABVÁNY MSZ 11007: Étkezési só (Nátrium-klorid)]. Hungarian Standards Institution [Internet]. 2013 [cited 2025 Jul 18]. Available from: https://www.tejfalussy.com/files/msz11007szabvany_szabvanyterrorizmus.pdf

24. Nyström HF, Brantsæter AL, Erlund I, Gunnarsdottir I, Hulthén L, Laurberg P, et al. Iodine status in the Nordic countries – past and present. Food Nutr Res. 2016 Jan 8;60(1):31969.

25. Italy. Decree No. 562 Regulation concerning the production and commercialization of table salt and iodised salt [DECRETO 10 agosto 1995, n. 562 Regolamento concernente la produzione e il commercio di sale da cucina iodurato, di sale iodato e di sale iodurato e iodato]. Ministry of Health [Internet]. 1995 [cited 2025 Jul 17]. Available from: https://www.normattiva.it/uri-res/N2Ls?urn:nir:ministero.sanita:decreto:1995-08-10;562

26. Italy. Law 21 March 2005 No. 55 Provisions aimed at the prevention of endemic goitre and of other iodine deficiency diseases [LEGGE 21 marzo 2005, n. 55 Disposizioni finalizzate alla prevenzione del gozzo endemico e di altre patologie da carenza iodica]. President of the Republic [Internet]. 2005 [cited 2025 Jul 17]. Available from: https://www.normattiva.it/uri-res/N2Ls?urn:nir:stato:legge:2005-03-21;55!vig=

27. Latvia. Cabinet Regulation No. 488 Minimum safety, quality, hygiene and labelling requirements for edible salt and requirements for the distribution and use of salt in food production [Obligātās nekaitīguma, kvalitātes, higiēnas un marķējuma prasības pārtikā lietojamajam sālim un prasības sāls izplatīšanai un izmantošanai pārtikas ražošanā] [Internet]. 2005 [cited 2025 Jul 18]. Available from: https://www.vestnesis.lv/ta/id/112120-obligatas-nekaitiguma-kvalitates-higienas-un-markejuma-prasibas-partika-lietojamajam-salim-un-prasibas-sals-izplatisanai-un-izm...

28. Latvia. Cabinet Regulation No. 696 regarding edible salt [Internet]. 2015 [cited 2025 Jul 18]. Available from: https://likumi.lv/ta/en/en/id/278399-regulations-regarding-edible-salt

29. Switzerland. Regulation on the addition of essential or physiologically useful substances to foodstuffs [Verordnung des EDI über den Zusatz essenzieller oder physiologisch nützlicher Stoffe zu Lebensmitteln]. Federal Department of Home Affairs [Internet]. 2005 [cited 2025 Jul 16]. Available from: https://www.lexfind.ch/tolv/142437/de

30. Switzerland. Regulation on the addition of essential or physiologically useful substances to foodstuffs [Verordnung des EDI über den Zusatz von Vitaminen, Mineralstoffen und sonstigen Stoffen in Lebensmitteln]. Federal Department of Home Affairs [Internet]. 2016 [cited 2025 Jul 16]. Available from: https://www.lexfind.ch/tolv/230859/de

31. Lithuania. Amendment of the Lithuanian Hygiene Standard HN 15:2005 [ĮSAKYMAS DĖL LIETUVOS RESPUBLIKOS SVEIKATOS APSAUGOS MINISTRO 2005 M. RUGSĖJO 1 D. ĮSAKYMO NR. V-675 „DĖL LIETUVOS HIGIENOS NORMOS HN 15:2005 „MAISTO HIGIENA“ PATVIRTINIMO“ PAKEITIMO]. Minister of Health [Internet]. 2005. 2005 [cited 2025 Jul 18]. Available from: https://e-seimas.lrs.lt/portal/legalAct/lt/TAD/c5c3f660991111eb998483d0ae31615c

32. Netherlands. Commodities Act Decree on the addition of micronutrients to food [Warenwetbesluit Toevoeging micro-voedingsstoffen aan levensmiddelen] [Internet]. 1994 [cited 2025 Jul 16]. Available from: https://wetten.overheid.nl/BWBR0008065/1999-12-01

33. Netherlands. Commodities Act Decree on the addition of micronutrients to food [Warenwetbesluit Toevoeging micro-voedingsstoffen aan levensmiddelen] [Internet]. 2008 [cited 2025 Jul 16]. Available from: https://wetten.overheid.nl/BWBR0008065/2008-06-20

34. Netherlands. Commodities Act Decree on the addition of micronutrients to food [Warenwetbesluit Toevoeging micro-voedingsstoffen aan levensmiddelen] [Internet]. 2014 [cited 2025 Jul 16]. Available from: https://wetten.overheid.nl/BWBR0008065/2014-11-14

35. Norway. Regulation FOR-2010-02-26-247 on the addition of vitamins, minerals and certain substances to foodstuffs [Forskrift om tilsetning av vitaminer, mineraler og visse andre stoffer til næringsmidler]. Ministry of Health and Welfare [Internet]. 2010 [cited 2025 Jul 16]. Available from: https://lovdata.no/dokument/SF/forskrift/2010-02-26-247

36. Poland. Regulation of 19 December 2002 on enriching substances added to food and conditions of their use [Rozporządzenie Ministra Zdrowia z dnia 19 grudnia 2002 r. w sprawie substancji wzbogacających dodawanych do żywności i warunków ich stosowania]. Minister of Health [Internet]. 2002 [cited 2025 Jul 16]. Available from: https://sip.lex.pl/akty-prawne/dzu-dziennik-ustaw/substancje-wzbogacajace-dodawane-do-zywnosci-i-warunki-ich-stosowania-17007029

37. Poland. Regulation of 16 September 2010 on enriching substances added to food and conditions of their use  [Rozporządzenie Ministra Zdrowia z dnia 16 września 2010 r. w sprawie substancji wzbogacających dodawanych do żywności]. Minister of Health [Internet]. 2010 [cited 2025 Jul 16]. Available from: https://eli.gov.pl/eli/DU/2010/1184/ogl/pol

38. Poland. Regulation of 13 March 2024 on enriching substances added to food and conditions of their use  [Rozporządzenie Ministra Zdrowia z dnia 13 marca 2024 r. w sprawie substancji wzbogacających dodawanych do żywności]. Minister of Health [Internet]. 2024 [cited 2025 Jul 16]. Available from: https://sip.lex.pl/akty-prawne/dzu-dziennik-ustaw/substancje-wzbogacajace-dodawane-do-zywnosci-21955487

39. Portugal. Decree-Law no. 87/96, Official Gazette Series I-A, no. 152 [Decreto-Lei n.o 87/96, de 3 de Julho 1986]. Ministry of Health [Internet]. 1996 [cited 2025 Jul 18]. Available from: https://files.dre.pt/1s/1996/07/152a00/17081709.pdf

40. Portugal. Circular no. 3/DSEEAS/DGE/2013 School guidelines – 2013/2014 [ Circular no 3/DSEEAS/DGE/2013 Orientacõórios  escolares – 2013/2014]. Ministry of Education [Internet]. 2013 [cited 2025 Jul 18]. Available from: https://www.dgeste.mec.pt/wp-content/uploads/2014/01/ASE_circular_3.pdf

41. Portugal. Guidelines on menus and school canteens [Orientações Sobre Ementas e Refeitórios Escolares]. Ministry of Education [Internet]. 2018 [cited 2025 Jul 18]. Available from: https://www.dge.mec.pt/sites/default/files/Esaude/oere.pdf

42. Romania. Decision No. 568/2002 on the universal iodization of salt for  human consumption, animal feed and use in the food industry [Hotărârea nr. 568/2002 privind iodarea universală a sării destinate consumului uman, hranei animalelor şi utilizării în industria alimentară]. Government of Romania [Internet]. 2002 [cited 2025 Jul 18]. Available from: https://legislatie.just.ro/Public/DetaliiDocument/36611

43. Romania. Decision No 473 of 1 April 2004 amending Government Decision No 568/2002 on the universal iodization of salt intended for human consumption, animal feed and use in the food industry [Hotărâre nr. 473 din 1 aprilie 2004 pentru modificarea Hotărârii Guvernului nr. 568/2002 privind iodarea universală a sării destinate consumului uman, hranei animalelor şi utilizării în industria alimentară]. Government of Romania [Internet]. 2004 [cited 2025 Jul 18]. Available from: https://legislatie.just.ro/Public/DetaliiDocumentAfis/51326

44. Romania. Decision No. 1.904 of 22 December 2006 amending Government Decision No. 568/2002 on the universal iodization of salt intended for human consumption, animal feed and use in the food industry [Hotărâre nr. 1.904 din 22 decembrie 2006 pentru modificarea Hotărârii Guvernului nr. 568/2002 privind iodarea universală a sării destinate consumului uman, hranei pentru animale şi utilizării în industria alimentară]. Government of Romania [Internet]. 2006 [cited 2025 Jul 18]. Available from: https://legislatie.just.ro/Public/DetaliiDocumentAfis/78040

45. Slovakia. Decree 309/2015 on snacks, edible salt, dehydrated dishes, soups and flavourings [Vyhláška Ministerstva pôdohospodárstva a rozvoja vidieka Slovenskej republiky o pochutinách, jedlej soli, dehydrovaných pokrmoch, polievkových prípravkoch a o ochucovadlách]. Ministry of Agriculture and Rural Development [Internet]. 2015 [cited 2025 Jul 18]. Available from: https://www.zakonypreludi.sk/zz/2015-309

46. Žmitek K, Pravst I. Iodisation of salt in Slovenia: Increased availability of non-iodised salt in the food supply. Nutrients [Internet]. 2016 Jul 16 [cited 2025 Jul 18];8(7). Available from: https://www.mdpi.com/2072-6643/8/7/434

47. Slovenia. 2659 Rules on tea, mustard, table salt, baking powder, pudding powder [2659. Pravilnik o čaju, gorčici, jedilni soli, pecilnem prašku, prašku za puding in vanilijevem sladkorju, stran 7190]. Minister of Health [Internet]. 1999 [cited 2025 Jul 18]. Available from: https://www.uradni-list.si/glasilo-uradni-list-rs/vsebina/20639

48. Slovenia. 3397 Rules on the quality of salt. Official Gazette of the Republic of Slovenia [3397 Pravilnik o kakovosti soli]. Minister of Agriculture, Forestry and Food [Internet]. 2003 [cited 2025 Jul 18]. Available from: https://www.uradni-list.si/glasilo-uradni-list-rs/vsebina/44331

49. Spain. Royal Decree 1424/1983, of April 27, approving the technical sanitary regulation for obtaining, circulating and selling of salt and edible brines [Real Decreto 1424/1983, de 27 de abril, por el que se aprueba la Reglamentación Técnico-Sanita ria para la obtención, circulación y venta de la sal y salmueras comestibles]. Presidency of the Government of Spain [Internet]. 1983 [cited 2025 Jul 18]. Available from: https://www.boe.es/buscar/act.php?id=BOE-A-1983-15544

50. Spain. Protocol of minimum criteria for the evaluation of food supply in schools: school canteens, food and beverage vending machines and cafeterias 2021–2025 [Protocolo de criterios mínimos para la evaluación de  la oferta alimentaria en centros escolares:  comedores escolares, máquinas expendedoras  de alimentos y bebidas y cafeter í as de acuerdo  al programa 16 del plan nacional de control oficial de  la cadena alim entaria 2021–2025]. Ministry of Consumption [Internet]. 2022 [cited 2025 Jul 18]. Available from: https://www.aesan.gob.es/AECOSAN/docs/documentos/nutricion/entorno_escolar/programa_16_protocolo.pdf

51. Sweden. Swedish Regulation: SLVFS 1983:2  (Amendment: LIVSFS 2002:19) Regulations on enrichment of certain foods [Föreskrifter om ändring i Livsmedelsverkets föreskrifter (SLVFS 1983:2) om berikning av vissa livsmedel] [Internet]. Sweden; 2002 [cited 2025 Jul 16]. Available from: https://www.livsmedelsverket.se/globalassets/om-oss/lagstiftning/berikn---kosttillsk---livsm-spec-gr-fsmp/livsfs-2002-19.pdf?siteid:67f9c486-281d-4765-ba72-ba3914739e3b,andquerymatch

52. Sweden. Amendment LIVSFS 2007:9 to Regulation SLVFS 1983:2 on the enrichment of certain foods [Livsmedelsverkets föreskrifter om berikning av vissa livsmedel] [Internet]. 2007 [cited 2025 Jul 18]. Available from: https://www.livsmedelsverket.se/globalassets/om-oss/lagstiftning/berikn---kosttillsk---livsm-spec-gr-fsmp/livsfs-2007-9.pdf

53. Türkiye. Turkish Food Codex Salt Communique No. 2007/53. Ministry of Food, Agriculture and Livestock [Internet]. 2008 [cited 2025 Jul 18]. Available from: https://faolex.fao.org/docs/pdf/tur110104.pdf

54. Türkiye. Turkish Food Codex Salt Communique [Türk gida kodeksġ tuz teblġğg]. Food, Agriculture and Livestock Minister [Internet]. 2013 [cited 2025 Jul 18]. Available from: https://faolex.fao.org/docs/pdf/tur150340.pdf

55. Ukraine. Iodized Salt – Technical Specifications (DSTU 4307:2004) [Сіль йодована – Технічні умови (ДСТУ 4307:2004)]. State Consumer Standard of Ukraine [Internet]. 2004 [cited 2025 Jul 18]. Available from: https://www.ksv.biz.ua/GOST/DSTY_ALL/DSTY2/dsty_4307-2004.pdf

56. Bath SC, Button S, Rayman MP. Availability of iodised table salt in the UK – is it likely to influence population iodine intake? Public Health Nutr. 2014 Feb 16;17(2):450–4.
