# Supplementary material for: Hidden hunger in Europe: a review on determinants, fragmented policy responses, and implementation barriers
Source: Front Nutr. 2025 Oct 20;12:1669008. doi: 10.3389/fnut.2025.1669008 (PMC12580594; doi:10.3389/fnut.2025.1669008)
Supplement: Supplementary file 2 [file Table_2.docx]

**Supplementary Table 2 and Sources: National food fortification policies in the EEA region**

| **Country** | **Regulation & reference** | **Year** | **Approach** | **Food vehicles** | **Micronutrients** | **Nutrient values** |
| --- | --- | --- | --- | --- | --- | --- |
| **Austria** | Federal Minister of Health and Consumer Protection. Regulation No. 531/1995 on infant formula and follow-on formula. 1995 (1) | 1995 (repealed) | Mandatory | Infant formula | Vitamin A | 60–180 µg retinol equivalents/100 kcal |
|  |  |  |  |  | Vitamin D | 1–2.5 µg/100 kcal |
|  |  |  |  |  | Thiamine | Min 40 µg/100 kcal |
|  |  |  |  |  | Riboflavin | Min 60 µg/100 kcal |
|  |  |  |  |  | Nicotinic acid amide | Min 250 µg niacin equivalents/100 kcal |
|  |  |  |  |  | Pantothenic acid | Min 300 µg/100 kcal |
|  |  |  |  |  | Vitamin B6 | Min 35 mg/100 kcal |
|  |  |  |  |  | Biotin | Min 1.5 µg/100 kcal |
|  |  |  |  |  | Folic acid | Min 4 µg/100 kcal |
|  |  |  |  |  | Vitamin B12 | Min 0.1 µg/100 kcal |
|  |  |  |  |  | Vitamin C | Min 8 mg/100 kcal |
|  |  |  |  |  | Vitamin K | Min 4 µg /100 kcal |
|  |  |  |  |  | Vitamin E | Min 0.5 mg α-tocopherol equivalents/100 kcal |
|  |  |  |  |  | Sodium | 20–60 mg/100 kcal |
|  |  |  |  |  | Potassium | 60–145 mg/100 kcal |
|  |  |  |  |  | Chlorine | 50–125 mg/100 kcal |
|  |  |  |  |  | Calcium | Min 50 mg/100 kcal |
|  |  |  |  |  | Phosphor | 25–90 mg/100 kcal |
|  |  |  |  |  | Magnesium | 5–15 mg/100 kcal |
|  |  |  |  |  | Iron (in formula made exclusively from cow milk protein) | 0.5–1.5 mg/100 kcal |
|  |  |  |  |  | Iron (in formula containing soy protein isolates) | 1–2 mg/100 kcal |
|  |  |  |  |  | Zinc (in formula made exclusively from cow milk protein) | 0.5–1.5 mg/100 kcal |
|  |  |  |  |  | Zinc (in formula containing soy protein isolates) | 0.75–2.4 mg/kcal |
|  |  |  |  |  | Copper | 20–80 µg/100 kcal |
|  |  |  |  |  | Iodine | Min 5 µg/100 kcal |
|  |  |  |  | Follow-on formula | Vitamin A | 60–180 µg retinol equivalents/100 kcal |
|  |  |  |  |  | Vitamin D | 1–3 µg/100 kcal |
|  |  |  |  |  | Vitamin C | Min 8 mg/100 kcal |
|  |  |  |  |  | Vitamin E | Min 0.5 mg α-tocopherol equivalents/100 kcal |
|  |  |  |  |  | Iron | 1–2 mg/100 kcal |
|  |  |  |  |  | Iodine | Min 5 µg/100 kcal |
|  |  |  |  |  | Zinc (formula made exclusively from cow’s milk) | Min 0.12 mg/100 kcal |
|  |  |  |  |  | Zinc (formula containing soy protein isolates) | Min 0.75 mg/100 kcal |
|  | Federal Minister of Health, Family and Youth. Regulation No. 68/2008 on infant formula and follow-on formula. 2008 (2) | 2008 | Mandatory | Infant formula | The only addition to the previous legislation:  Selenium (in infant formula exclusively made from cow milk) | Max 9 µg/100 kcal |
| **Belgium** | Royal Decree on the manufacture and marketing of margarine and edible fats. 1980 (3) | 1980 | Mandatory | Margarine, edible fats | Vitamin A | 750–900 µg/100 g |
|  |  |  |  |  | Vitamin D | 6.25–7.5 µg/100 g |
| **Finland** | Ministry of Agriculture and Forestry. Decree 182/1987 on the vitaminization of certain food fats and on the use of additives and identification substances in these products. 1987 (4) | 1987 | Voluntary | Margarine, butter-vegetable oil mixtures and fat mixtures | Vitamin A/ Beta-carotene | 500–1000 µg/100 g |
|  |  |  |  |  | Vitamin D | 5–10 µg/100 g |
|  | Ministry of Trade and Industry. Decree 917/2002 on the addition of vitamins and certain other substances to foodstuffs. 2002 (5) (updated 2010 (6)) | 2002 (updated 2010) | Voluntary | Whole juice, juice, nectar and similar concentrates | Vitamin C | 25–60 mg/100 ml |
|  |  |  |  |  | Calcium | 120 mg/100 ml |
|  |  |  |  | Berry and fruit-based desserts | Vitamin C | 25–60 mg/100 g |
|  |  |  |  | Mashed potato products | Vitamin C | 25–60 mg/100 g |
|  |  |  |  | Lozenges and chewing gum | Vitamin C | 50–500 mg/100 g |
|  |  |  |  | Breakfast cereals | Thiamine | 1–1.4 mg/100 g |
|  |  |  |  |  | Riboflavin | 1–1.8 mg/100 g |
|  |  |  |  |  | Niacin | 15–17 mg/100 g |
|  |  |  |  |  | Iron | 6.5 mg/100 g |
|  |  |  |  | Spreadable fats | Vitamin A | 500–1000 µg/100 g |
|  |  |  |  |  | Beta-carotene | 500–1000 µg calculated as retinol/100 g |
|  |  |  |  |  | Vitamin D | 10 µg/100 g (20 µg/100 g since 2010) |
|  |  |  |  | Milk, liquid dairy products, lactose-free milk, soy and grain-based | Vitamin D | 0.5 µg/100 ml (1 µg/100 ml since 2010) |
|  |  |  |  | Liquid preparations | Calcium | 120 mg/100 ml |
|  | Ministry of Agriculture and Forestry. Decree 754/2016 (7) on vitamin D fortification of skimmed homogenized milk. 2016 | 2016 | Mandatory | Skimmed homogenized milk | Vitamin D | Minimum 1 µg/100 ml |
| **Germany** | No formal legislation on food fortification has been identified. | | | | | |
| **Greece** | Ministry of Rural Development and Food. Ministerial Decree No. 1100/1987) codifying the provisions of the Food Code. 1987 (8) | 1987–present | Voluntary | No explicit food vehicle or micronutrient values could be retrieved from the available sources. | | |
| **Hungary** | No formal legislation on food fortification has been identified. | | | | | |
| **Ireland** | No formal legislation on food fortification has been identified. | | | | | |
| **Liechtenstein** | Federal Department of Home Affairs (FDHA). Regulation on the addition of essential or physiologically useful substances to foodstuffs. 2005 (9) (repealed 2016) | 2005 (repealed) | Voluntary | Broad food categories listed in *Annex 3* | Vitamins and minerals in *Annex 1* | The addition must be such that the recommended daily dose (*Annex 1*) is not exceeded through the daily ration (*Annex 3*). |
|  | FDHA Ordinance on the addition of vitamins, minerals and other substances in foodstuffs. 2016 (10) (latest version 2023) | 2016 | Voluntary | Similar range of food vehicles in *Annex 7*, including added milk-based beverages and fish products. | Micronutrients allowed listed in *Annex 1.* Adds new ones (phosphorus, copper, manganese, chromium, molybdenum, chloride). | The addition of nutrients must comply with the maximum levels listed in *Annex 1* per daily ration defined in *Annex 7*. |
| **Netherlands (the Kingdom of)** | Commodities Act Decree on the addition of micronutrients to food. 1999 (11) (valid until 2004) | 1999–2004 | Voluntary | General fortified food (not substitution or restoration) | Micronutrients listed in *Annex 2* (vitamin A, B1, B2, niacin, B6, pantothenic acid, B12, biotin, C, E, K) & *Annex 3* (calcium, magnesium, iron, manganese, phosphorus, chromium, molybdenum, sodium, potassium, chlorine) | Must provide 15–100% of the RDI per reasonable daily consumption. |
|  |  |  |  |  | Other allowed micronutrients from Annex 1 not listed in *Annex 2* or *Annex 3* (copper, zinc, selenium, chromium, phosphorus) | No specific concentration limits |
|  |  |  |  | Substitution and restoration products | Vitamin A, vitamin D, folic acid, selenium, copper, zinc | Allowed only for substitution/restoration; may match natural content in original product |
|  |  |  |  | Spreadable fats | Vitamin A | Max 800 µg/100 g |
|  |  |  |  |  | Vitamin D | Max 7.5 µg/100 g |
|  | Commodities Act Decree on the addition of micronutrients to food. 2008 (12) (valid until 2010) | 2008–2010 | Voluntary | General fortified food | Micronutrients listed in *Annex 2* and *Annex 3* (same as in the previous legislation). | 15–100% of the RDI |
|  |  |  |  |  | Other allowed micronutrients from Annex 1 not listed in *Annex 2* or *Annex 3* (selenium). | No specific concentration limits |
|  |  |  |  | Substitution and restoration products | Same as in the previous legislation | |
|  |  |  |  | Spreadable fats | Same as in the previous legislation | |
|  | Commodities Act Decree on the addition of micronutrients to food. 2014 (13) | 2014–present | Voluntary | General fortified food | Micronutrients listed in *Annex 2* & *Annex 3* | 15–100% of the RDI |
|  |  |  |  | Substitution and restoration products | Same as in the previous legislation | |
|  |  |  |  | Spreadable fats | Same as in the previous legislation | |
| **Norway** | Ministry of Health and Welfare. Regulation FOR-2010-02-26-247 on the addition of vitamins, minerals and certain substances to foodstuffs. 2010 (14) | 2010 (amended 2025) | Voluntary | Bread | Vitamin D | Max 5 µg/100 g |
|  |  |  |  | Breakfast cereal | Niacin | Max 11 mg/100 g |
|  |  |  |  |  | Vitamin B6 | Max 1.4 mg/100 g |
|  |  |  |  |  | Folic acid | Max 133 µg/100 g |
|  |  |  |  |  | Calcium | Max 459 mg/ 100 g |
|  |  |  |  | Gluten-free muesli/cereal | Niacin | Max 16 mg/100 g |
|  |  |  |  |  | Vitamin B6 | Max 1.4 mg/100 g |
|  |  |  |  |  | Folic acid | Max 183 µg/100 g |
|  |  |  |  |  | Magnesium | Max 121 mg/100 g |
|  |  |  |  |  | Iron | Max 5 mg/100 g |
|  |  |  |  | Gluten-free and low-protein bread/flour mixes | Niacin | Max 4 mg/100 g |
|  |  |  |  |  | Vitamin B6 | Max 2.5 mg/100 g |
|  |  |  |  |  | Folic acid | Max 40 µg/100 g |
|  |  |  |  |  | Iron | Max 9 mg/100 g |
|  |  |  |  | Gluten-free bread, crispbread and crusts | Niacin | Max 4 mg/100 g |
|  |  |  |  |  | Vitamin B6 | Max 2.5 mg/100 g |
|  |  |  |  |  | Folic acid | Max 40 µg/100 g |
|  |  |  |  |  | Iron | Max 9 mg/100 g |
|  |  |  |  | Milk (all types of milk sold directly to consumers) | Vitamin D | Max 1 µg/100 ml |
|  |  |  |  |  | Folic acid | Max 15 µg/100 ml |
|  |  |  |  | Milk-based beverages (including fermented and/or flavored) | Vitamin D | Max 1.9 µg/100 ml |
|  |  |  |  |  | Vitamin C | Max 11.3 mg/100 ml |
|  |  |  |  |  | Niacin | Max 3 mg/100 ml |
|  |  |  |  |  | Vitamin B6 | Max 0.29 mg/100 ml |
|  |  |  |  |  | Calcium | Max 120 mg/100 ml |
|  |  |  |  |  | Chrome | Max 5 µg/100 ml |
|  |  |  |  | Condensed milk | Vitamin D | Max 1.1 µg/100 ml |
|  |  |  |  | Vegetable alternatives to milk-based beverages | Vitamin D | Max 1.5 µg/100 ml |
|  |  |  |  |  | Vitamin E | Max 1.8 mg/100 ml |
|  |  |  |  |  | Folic acid | Max 23 µg/100 ml |
|  |  |  |  |  | Calcium | Max 120 mg/100 ml |
|  |  |  |  |  | Iodine | Max 22.5 µg/100 ml |
|  |  |  |  | Fermented products such as yoghurt (does not include beverages) | Vitamin D | Max 2.9 µg/100 g |
|  |  |  |  |  | Vitamin C | Max 26 mg/100 g |
|  |  |  |  |  | Niacin | Max 2.4 mg/100 g |
|  |  |  |  |  | Vitamin B6 | Max 0.21 mg/100 g |
|  |  |  |  |  | Calcium | Max 120 mg/100 g |
|  |  |  |  | Vegetable alternatives to fermented milk products (does not include beverages) | Vitamin D | Max 1.5 µg/100 g |
|  |  |  |  |  | Vitamin E | Max 1.8 mg/100 g |
|  |  |  |  |  | Vitamin C | Max 13.3 mg/100 g |
|  |  |  |  |  | Calcium | Max 146 mg/100 g |
|  |  |  |  |  | Iodine | Max 22.5 µg/100 g |
|  |  |  |  | White cheese | Vitamin D | Max 4.1 µg/100 g |
|  |  |  |  |  | Vitamin E | Max 38 mg/100 g |
|  |  |  |  | Prime cheese | Vitamin D | Max 2.7 µg/100 g |
|  |  |  |  | Brunost and primes products for children | Vitamin D | Max 2.7 µg/100 g |
|  |  |  |  |  | Iron | Max 10 mg/100 g |
|  |  |  |  | Vegetable alternative to cheese | Vitamin D | Max 5 µg/100 g |
|  |  |  |  |  | Calcium | Max 664 mg/100 g |
|  |  |  |  |  | Iodine | Max 31 µg/100 g |
|  |  |  |  | Margarine, other types of edible fats and industrial shortening | Vitamin A | Max 900 µg/100 g |
|  |  |  |  |  | Vitamin D | Max 20 µg/100 g |
|  |  |  |  | Butter | Vitamin D | Max 10 µg/100 g |
|  |  |  |  | Cooking oil | Vitamin D | Max 10 µg/100 g |
|  |  |  |  |  | Vitamin E | Max 13 mg/100 g |
|  |  |  |  | Caviar on a tube | Vitamin D | Max 9.5 µg/100 g |
|  |  |  |  |  | Vitamin E | Max 4.7 µg/100 g |
|  |  |  |  | Products used as alternative to fish | Iron | Max 2.1 µg/100 g |
|  |  |  |  | Liver pâté | Vitamin D | Max 2.5 µg/100 g |
|  |  |  |  |  | Vitamin E | Max 2.5 mg/100 g |
|  |  |  |  | Products used as an alternative to meat | Iron | Max 8.5 mg/100 g |
|  |  |  |  | Vegetable alternative to pâté | Iron | Max 5.9 mg/100 g |
|  |  |  |  | Fruit juice, vegetable juice, nectar, smoothies | Beta-carotene | Max 120 µg/100 ml |
|  |  |  |  |  | Vitamin D | Max 2.5 µg/100 ml |
|  |  |  |  |  | Vitamin E | Max 4 mg/100 ml |
|  |  |  |  |  | Vitamin C | Max 96 mg/100 ml |
|  |  |  |  |  | Niacin | Max 3.2 mg/100 g |
|  |  |  |  |  | Vitamin B6 | Max 0.3 mg/100 ml |
|  |  |  |  |  | Folic acid | Max 30 µg/100 ml |
|  |  |  |  |  | Magnesium | Max 45 mg/100 ml |
|  |  |  |  | Water-based non-alcoholic beverages with and without carbonation | Vitamin D | Max 0.89 µg/100 ml |
|  |  |  |  |  | Vitamin E | Max 3.7 mg/100 ml |
|  |  |  |  |  | Vitamin C | Max 40 mg/100 ml |
|  |  |  |  |  | Niacin | Max 8 mg/100 ml |
|  |  |  |  |  | Vitamin B6 | Max 1.4 mg/100 ml |
|  |  |  |  |  | Folic acid | Max 40 µg/100 ml |
|  |  |  |  |  | Calcium | Max 60 mg/100 ml |
|  |  |  |  |  | Magnesium | Max 29 mg/100 ml |
|  |  |  |  |  | Selenium | Max 0.15 µg/100 ml |
|  |  |  |  | Water-based non-alcoholic beverages with and without carbonation with added caffeine in an amount exceeding 15 mg/100 ml ^​^ | Vitamin D | Max 1.2 µg/100 ml |
|  |  |  |  |  | Vitamin E | Max 4 mg/100 ml |
|  |  |  |  |  | Vitamin C | Max 48 mg/100 ml |
|  |  |  |  |  | Niacin | Max 10 mg/100 ml |
|  |  |  |  |  | Vitamin B6 | Max 1.4 mg/100 ml |
|  |  |  |  |  | Folic acid | Max 24.4 µg/100 ml |
|  |  |  |  |  | Calcium | Max 60 mg/100 ml |
|  |  |  |  |  | Magnesium | Max 30 mg/100 ml |
|  |  |  |  |  | Zinc | Max 2 mg/100 ml |
|  |  |  |  | Water-based non-alcoholic beverages with and without carbonation portioned in smaller drinking ampoules, shots or similar | Niacin | Max 1.5 mg/100 ml |
|  |  |  |  |  | Vitamin B6 | Max 2.3 mg/100 ml |
|  |  |  |  |  | Folic acid | Max 15 µg/100 ml |
|  |  |  |  | Electrolyte drinks | Vitamin A | Max 290 µg/100 ml |
|  |  |  |  |  | Vitamin D | Max 2.5 µg/100 ml |
|  |  |  |  |  | Vitamin E | Max 4.5 mg/100 ml |
|  |  |  |  |  | Vitamin C | Max 35 mg/100 ml |
|  |  |  |  |  | Niacin | Max 7.5 mg/100 ml |
|  |  |  |  |  | Vitamin B6 | Max 0.9 mg/100 ml |
|  |  |  |  |  | Folic acid | Max 82 µg/100 ml |
|  |  |  |  |  | Calcium | Max 180 mg/100 ml |
|  |  |  |  |  | Phosphorus | Max 150 mg/100 ml |
|  |  |  |  |  | Magnesium | Max 60 mg/100 ml |
|  |  |  |  |  | Iron | Max 3 mg/100 ml |
|  |  |  |  |  | Zinc | Max 1.5 mg/100 ml |
|  |  |  |  |  | Copper | Max 0.2 mg/100 ml |
|  |  |  |  |  | Manganese | Max 0.4 mg/100 ml |
|  |  |  |  |  | Selenium | Max 8.5 µg/100 ml |
|  |  |  |  |  | Chrome | Max 20 µg/100 ml |
|  |  |  |  |  | Molybdenum | Max 17 µg/100 ml |
|  |  |  |  |  | Iodine | Max 23 µg/100 ml |
|  |  |  |  | Carbohydrate-electrolyte drinks that also contain protein/fat ^​^of which the protein content constitutes at least 20% of the energy content of the product and the energy content is at least 420 kJ / 100 ml (100 kcal / 100 ml) | Vitamin A | Max 125 µg/100 ml |
|  |  |  |  |  | Vitamin D | Max 2 µg/100 ml |
|  |  |  |  |  | Vitamin E | Max 2.2 mg/100 ml |
|  |  |  |  |  | Vitamin C | Max 32 mg/100 ml |
|  |  |  |  |  | Niacin | Max 2.5 mg/100 ml |
|  |  |  |  |  | Vitamin B6 | Max 0.6 mg/100 ml |
|  |  |  |  |  | Folic acid | Max 35 µg/100 ml |
|  |  |  |  |  | Calcium | Max 210 mg/100 ml |
|  |  |  |  |  | Phosphorus | Max 175 mg/100 ml |
|  |  |  |  |  | Magnesium | Max 60 mg/100 ml |
|  |  |  |  |  | Iron | Max 2.6 mg/100 ml |
|  |  |  |  |  | Zinc | Max 1.5 mg/100 ml |
|  |  |  |  |  | Copper | Max 0.3 mg/100 ml |
|  |  |  |  |  | Manganese | Max 0.3 mg/100 ml |
|  |  |  |  |  | Selenium | Max 8.5 µg/100 ml |
|  |  |  |  |  | Molybdenum | Max 17 µg/100 ml |
|  |  |  |  |  | Iodine | Max 23 µg/100 ml |
|  |  |  |  | Meal replacements for weight control (products that are labelled and marketed as a replacement for 1–2 main meals) | Vitamin A | Max 491 µg/100 g |
|  |  |  |  |  | Vitamin D | Max 4.7 µg/100 g |
|  |  |  |  |  | Vitamin E | Max 8.7 mg/100 g |
|  |  |  |  |  | Vitamin K | Max 36 µg/100 g |
|  |  |  |  |  | Vitamin C | Max 53 mg/100 g |
|  |  |  |  |  | Niacin | Max 11 mg/100 g |
|  |  |  |  |  | Vitamin B6 | Max 1.6 mg/100 g |
|  |  |  |  |  | Folic acid | Max 147 mg/100 g |
|  |  |  |  |  | Calcium | Max 458 mg/100 g |
|  |  |  |  |  | Phosphorus | Max 552 mg/100 g |
|  |  |  |  |  | Magnesium | Max 157 mg/100 g |
|  |  |  |  |  | Iron | Max 11 mg/100 g |
|  |  |  |  |  | Zinc | Max 5 mg/100 g |
|  |  |  |  |  | Copper | Max 0.85 mg/100 g |
|  |  |  |  |  | Manganese | Max 1.5 mg/100 g |
|  |  |  |  |  | Selenium | Max 40 µg/100 g |
|  |  |  |  |  | Chrome | Max 35 µg/100 g |
|  |  |  |  |  | Molybdenum | Max 31 µg/100 g |
|  |  |  |  |  | Iodine | Max 100 µg/100 g |
|  |  |  |  | Milk-based drinks (1–3 years) | Vitamin A | Max 500 µg/100 ml |
|  |  |  |  |  | Vitamin D | Max 9.0 µg/100 ml |
|  |  |  |  |  | Vitamin E | Max 11 mg/100 ml |
|  |  |  |  |  | Vitamin K | Max 45 µg/100 ml |
|  |  |  |  |  | Vitamin C | Max 110 mg/100 ml |
|  |  |  |  |  | Niacin | Max 5.0 mg/100 ml |
|  |  |  |  |  | Vitamin B6 | Max 0.53 mg/100 ml |
|  |  |  |  |  | Folic acid | Max 145 µg/100 ml |
|  |  |  |  |  | Calcium | Max 910 mg/100 ml |
|  |  |  |  |  | Phosphorus | Max 366 mg/100 ml |
|  |  |  |  |  | Magnesium | Max 57 mg/100 ml |
|  |  |  |  |  | Iron | Max 9.5 mg/100 ml |
|  |  |  |  |  | Zinc | Max 6 mg/100 ml |
|  |  |  |  |  | Copper | Max 0.38 mg/100 ml |
|  |  |  |  |  | Manganese | Max 77 µg/100 ml |
|  |  |  |  |  | Fluorine | Max 54 µg/100 ml |
|  |  |  |  |  | Selenium | Max 12 µg/100 ml |
|  |  |  |  |  | Iodine | Max 120 µg/100 ml |
|  |  |  |  | Mayonnaise | Vitamin D | Max 8 µg/100 g |
|  |  |  |  |  | Vitamin E | Max 4 mg/100 g |
|  |  |  |  |  | Chrome | Max 10 µg/100 g |
|  |  |  |  | Gels marketed in single-serving packages | Niacin | Max 9.6 mg/100 g |
|  |  |  |  |  | Vitamin B6 | Max 2.3 mg/100 g |
|  |  |  |  | Bars | Vitamin A | Max 433 µg/100 g |
|  |  |  |  |  | Vitamin D | Max 3.6 µg/100 g |
|  |  |  |  |  | Vitamin E | Max 14 mg/100 g |
|  |  |  |  |  | Vitamin C | Max 85 mg/100 g |
|  |  |  |  |  | Niacin | Max 21 mg/100 g |
|  |  |  |  |  | Vitamin B6 | Max 2.8 mg/100 g |
|  |  |  |  |  | Folic acid | Max 429 µg/100 g |
|  |  |  |  |  | Calcium | Max 428 mg/100 g |
|  |  |  |  |  | Phosphorus | Max 604 mg/100 g |
|  |  |  |  |  | Magnesium | Max 200 mg/100 g |
|  |  |  |  |  | Iron | Max 11 mg/100 g |
|  |  |  |  |  | Zinc | Max 6.2 mg/100 g |
|  |  |  |  |  | Copper | Max 0.83 mg/100 g |
|  |  |  |  |  | Manganese | Max 1.1 mg/100 g |
|  |  |  |  |  | Selenium | Max 38 µg/100 g |
|  |  |  |  |  | Chrome | Max 40 µg/100 g |
|  |  |  |  |  | Molybdenum | Max 50 µg /100 g |
|  |  |  |  |  | Iodine | Max 82.5 µg/100 g |
|  |  |  |  | Vegetable alternatives to dairy-based desserts | Vitamin D | Max 1.5 µg/100 g |
|  |  |  |  |  | Calcium | Max 120 mg/100 g |
|  |  |  |  |  | Iodine | Max 22.5 µg/100 g |
|  |  |  |  | Coffee powder (freeze-dried) | Chrome | Max 60 µg/100 g |
|  |  |  |  | Tea (stewed in water) | Vitamin C | Max 10 mg/100 g |
|  |  |  |  |  | Niacin | Max 1.2 mg/100 g |
|  |  |  |  |  | Vitamin B6 | Max 0.18 mg/100 g |
|  |  |  |  |  | Zinc | Max 0.75 mg/100 g |
|  |  |  |  |  | Selenium | Max 6.9 µg/100 g |
|  |  |  |  | Non-alcoholic beverages based on milk and coffee | Niacin | Max 6.4 mg/100 g |
|  |  |  |  |  | Vitamin B6 | Max 0.56 mg/100 g |
|  |  |  |  | Vegetable alternatives to "iced coffee latte" | Vitamin D | Max 0.75 µg/100 g |
|  |  |  |  |  | Vitamin E | Max 1.8 mg/100 g |
|  |  |  |  |  | Calcium | Max 120 mg/100 g |
|  |  |  |  | Fluoride tablets | Fluorine | Max 2.3 mg/100 g |
|  |  |  |  | Throat lozenges | Vitamin C | Max 215 mg/100 g |
|  |  |  |  |  | Zinc | Max 12.3 mg/100 g |
|  |  |  |  | Chewing gum | Vitamin C | Max 450 mg/100 g |
|  |  |  |  |  | Calcium | Max 1300 mg/100 g |
|  |  |  |  |  | Zinc | Max 24.3 mg/100 g |
|  |  |  |  |  | Fluorine | Max 10 mg/100 g |
|  |  |  |  | Wine gum | Niacin | Max 4 mg/100 g |
| **Poland** | Minister of Health. Regulation on enriching substances added to food and conditions of their use. 2002 (15) | 2002 (repealed) | Mandatory | Regular and reduced-fat margarine, reduced-fat butter and butter-oil mixtures | Vitamin A | Max 900 µg/100 g |
|  |  |  |  |  | Vitamin D | Max 7.5 µg/100 g |
|  |  |  | Voluntary | Edible vegetable oil | Vitamin E | 15–50% of the RDI |
|  |  |  |  | Margarine | Vitamin E | 15–50% of the RDI |
|  |  |  |  | Concentrate of powdered beverages containing dried fruit; dessert concentrate with dried fruit; seasoning for beverages with fruit juice | Vitamin A, vitamin D, thiamine, riboflavin, niacin, vitamin B6, vitamin B12, pantothenic acid, biotin | 15–50% of the RDI |
|  |  |  |  |  | Folate, vitamin C | 15–100% of the RDI |
|  |  |  |  | Milk and milk products with reduced fat content; fruit, vegetable, fruit and vegetable juices and nectars; beverages containing fruit and vegetable juices; flour and other cereal products; cereal breakfast products | Vitamin A, vitamin D, thiamine, riboflavin, niacin, vitamin B6, vitamin B12, pantothenic acid, biotin, calcium, iron | 15–50% of the RDI |
|  |  |  |  |  | Folate, vitamin C | 15–100% of the RDI |
|  | Minister of Health. Regulation on enriching substances added to food and conditions of their use. 2010 (16) | 2010 (repealed) | Same as 2002 | | | |
|  | Minister of Health. Regulation on enriching substances added to food and conditions of their use and prohibited substances. 2024 (17) | 2024 | Same as 2002/2010 | | | |
| **Sweden** | Swedish Government. Regulation SLVFS 1983:2 (18) on the fortification of certain foods (amended by LIVSFS 2002:9 (18) and LIVFSFS 2007:9 (19)) | 1983 (amended 2002 and 2007, repealed 2018) | Mandatory | Milk for direct consumption (<1.5% fat) | Vitamin D | 0.38–0.5 µg/100 ml |
|  |  |  |  | Margarine and fat mixtures | Vitamin A | 900–1500 µg/100 g |
|  |  |  |  |  | Vitamin D | 7.5–10 µg/100 g |
|  | Swedish Government. Regulation LIVSFS 2018:5 (20) on the enrichment of certain foods. 2018 | 2018 | Mandatory | Milk for direct consumption (<3% fat), vegetable and lactose-free products (<3% fat) | Vitamin D | 0.95–1.1 µg/100 g |
|  |  |  |  | Fermented milk (<3% fat) and alternative vegetable products for fermented milk | Vitamin D | 0.75–1.1 µg/100 g |
|  |  |  |  | Margarine and fat mixtures | Vitamin A | 900–1500 µg/100 g |
|  |  |  |  |  | Vitamin D | 19.5–21 µg/100 g |
| **Switzerland** | Federal Department of Home Affairs (FDHA) Regulation on the addition of essential or physiologically useful substances to foodstuffs (repealed 2016) (9) | 2005 (repealed) | Voluntary | Broad food categories listed in *Annex 3* | Vitamins and minerals in *Annex 1* | The addition must be such that the recommended daily dose (*Annex 1*) is not exceeded through the daily ration (*Annex 3*). |
|  | FDHA Ordinance on the addition of vitamins, minerals and other substances in foodstuffs (latest version 2023) (10) | 2016 | Voluntary | Similar range of food vehicles in *Annex 7*, including added milk-based beverages and fish products. | Micronutrients allowed listed in *Annex 1.* Adds new ones (phosphorus, copper, manganese, chromium, molybdenum, chloride). | The addition of nutrients must comply with the maximum levels listed in *Annex 1* per daily ration defined in *Annex 7*. |
| **Türkiye** | Ministry of Food, Agriculture and Livestock. Turkish Food Codex Regulation on the addition of vitamins and minerals and of certain other substances to foods (2017 (21), amended 2020 (22)) | 2017 (amended 2020) | Voluntary | All food excluding unprocessed foods (fruits, vegetables, red meat, poultry, fish) and alcoholic beverages with more than 1.2% alcohol by volume | Vitamins and minerals (A, D, E, C, K, B group; calcium, phosphorus, iron, magnesium, zinc, iodine, selenium, copper, manganese, chromium, molybdenum, fluoride, potassium, chloride) | No minimum/maximum amounts mentioned |
| **United Kingdom** | Bread and Flour Regulations (1998) (23) | 1998 | Mandatory | White and brown wheat flour | Calcium (carbonate) | 235–390 mg/100 g |
|  |  |  |  |  | Iron | Min 1.65 mg/100 g |
|  |  |  |  |  | Thiamine | Min 0.24 mg/100 g |
|  |  |  |  |  | Niacin | Min 1.6 mg/100 g |
|  | Bread and Flour Regulations (amended 2024 (24)) | 2024 | Mandatory | White and brown wheat flour | Calcium (carbonate) | 300–455 mg/100 g |
|  |  |  |  |  | Iron | Min 2.1 mg/100 g |
|  |  |  |  |  | Thiamine | Min 0.24 mg/100 g |
|  |  |  |  |  | Niacin | Min 2.4 mg/100 g |
|  |  |  |  |  | Folic Acid | 0.25 µg/100 g |

# Sources

1. Austria. Regulation No. 531/1995 on infant formula and follow-on formula [Verordnung des Bundesministers für Gesundheit und Konsumentenschutz über Säuglingsanfangsnahrung und Folgenahrung]. Federal Minister of Health and Consumer Protection [Internet]. 1995 [cited 2025 Jul 16]. Available from: https://www.ris.bka.gv.at/Dokumente/BgblPdf/1995_531_0/1995_531_0.pdf

2. Austria. Regulation No. 68/2008 on infant formula and follow-on formula [Verordnung des Bundesministers für Gesundheit und Konsumentenschutz über Säuglingsanfangsnahrung und Folgenahrung]. Federal Minister of Health, Family and Youth [Internet]. 2008 [cited 2025 Jul 16]. Available from: https://www.ris.bka.gv.at/eli/bgbl/II/2008/68/20080220

3. Belgium. Royal Decree on the manufacture and marketing of margarine and edible fats [Arrêté royal relatif à la fabrication et a la mise dans le commerce de la margarine et des graisses comestibles] [Internet]. 1980 [cited 2025 Jul 16]. Available from: https://refli.be/fr/lex/1980100202

4. Finland. Decree 182/1987 on the vitaminisation of certain food fats and on the use of additives and identification substances in these products [Jord- och skogsbruksministeriets beslut om vitaminisering av vissa livsmedelsfetter samt om användning av tillsatsämnen och identifieringsämnen i dessa produkter]. Ministry of Agriculture and Forestry [Internet]. 1987 [cited 2025 Jul 16]. Available from: https://finlex.fi/sv/lagstiftning/forfattningssamling/1987/182

5. Finland. Decree 917/2002 on the addition of vitamins and certain other substances to foodstuffs [Kauppa- ja teollisuusministeriön asetus vitamiinien ja eräiden muiden aineiden lisäämisestä elintarvikkeisiin]. Ministry of Trade and Industry [Internet]. 2002 [cited 2025 Jul 16]. Available from: https://finlex.fi/fi/lainsaadanto/saadoskokoelma/2002/917

6. Finnish Food Authority. Food fortification with Vitamin D [Internet]. 2010 [cited 2025 Jul 18]. Available from: https://www.ruokavirasto.fi/en/foodstuffs/healthy-diet/national-nutrition-council/proposals-for-measures-and-statements/action-proposals-for-food-fortification/food-fortification-with-vitamin-d/

7. Finland. Decree 754/2016 on vitamin D fortification of skimmed homogenized milk [Maa- ja metsätalousministeriön asetus rasvattoman homogenoidun maidon D-vitaminoinnista]. Ministry of Agriculture and Forestry [Internet]. 2016 [cited 2025 Jul 16]. Available from: https://finlex.fi/en/legislation/2016/754

8. Greece. Ministerial Decree No 1100/1987 codifying the provisions of the Food Code. Ministry of Rural  Development and Food [Internet]. 1987 [cited 2025 Jul 16]. Available from: https://www.fao.org/faolex/results/details/en/c/LEX-FAOC106642/

9. Switzerland. Regulation on the addition of essential or physiologically useful substances to foodstuffs [Verordnung des EDI über den Zusatz essenzieller oder physiologisch nützlicher Stoffe zu Lebensmitteln]. Federal Department of Home Affairs [Internet]. 2005 [cited 2025 Jul 16]. Available from: https://www.lexfind.ch/tolv/142437/de

10. Switzerland. Regulation on the addition of essential or physiologically useful substances to foodstuffs [Verordnung des EDI über den Zusatz von Vitaminen, Mineralstoffen und sonstigen Stoffen in Lebensmitteln]. Federal Department of Home Affairs [Internet]. 2016 [cited 2025 Jul 16]. Available from: https://www.lexfind.ch/tolv/230859/de

11. Netherlands. Commodities Act Decree on the addition of micronutrients to food [Warenwetbesluit Toevoeging micro-voedingsstoffen aan levensmiddelen] [Internet]. 1994 [cited 2025 Jul 16]. Available from: https://wetten.overheid.nl/BWBR0008065/1999-12-01

12. Netherlands. Commodities Act Decree on the addition of micronutrients to food [Warenwetbesluit Toevoeging micro-voedingsstoffen aan levensmiddelen] [Internet]. 2008 [cited 2025 Jul 16]. Available from: https://wetten.overheid.nl/BWBR0008065/2008-06-20

13. Netherlands. Commodities Act Decree on the addition of micronutrients to food [Warenwetbesluit Toevoeging micro-voedingsstoffen aan levensmiddelen] [Internet]. 2014 [cited 2025 Jul 16]. Available from: https://wetten.overheid.nl/BWBR0008065/2014-11-14

14. Norway. Regulation FOR-2010-02-26-247 on the addition of vitamins, minerals and certain substances to foodstuffs [Forskrift om tilsetning av vitaminer, mineraler og visse andre stoffer til næringsmidler]. Ministry of Health and Welfare [Internet]. 2010 [cited 2025 Jul 16]. Available from: https://lovdata.no/dokument/SF/forskrift/2010-02-26-247

15. Poland. Regulation of 19 December 2002 on enriching substances added to food and conditions of their use [Rozporządzenie Ministra Zdrowia z dnia 19 grudnia 2002 r. w sprawie substancji wzbogacających dodawanych do żywności i warunków ich stosowania]. Minister of Health [Internet]. 2002 [cited 2025 Jul 16]. Available from: https://sip.lex.pl/akty-prawne/dzu-dziennik-ustaw/substancje-wzbogacajace-dodawane-do-zywnosci-i-warunki-ich-stosowania-17007029

16. Poland. Regulation of 16 September 2010 on enriching substances added to food and conditions of their use  [Rozporządzenie Ministra Zdrowia z dnia 16 września 2010 r. w sprawie substancji wzbogacających dodawanych do żywności]. Minister of Health [Internet]. 2010 [cited 2025 Jul 16]. Available from: https://eli.gov.pl/eli/DU/2010/1184/ogl/pol

17. Poland. Regulation of 13 March 2024 on enriching substances added to food and conditions of their use  [Rozporządzenie Ministra Zdrowia z dnia 13 marca 2024 r. w sprawie substancji wzbogacających dodawanych do żywności]. Minister of Health [Internet]. 2024 [cited 2025 Jul 16]. Available from: https://sip.lex.pl/akty-prawne/dzu-dziennik-ustaw/substancje-wzbogacajace-dodawane-do-zywnosci-21955487

18. Sweden. Swedish Regulation: SLVFS 1983:2  (Amendment: LIVSFS 2002:19) Regulations on enrichment of certain foods [Föreskrifter om ändring i Livsmedelsverkets föreskrifter (SLVFS 1983:2) om berikning av vissa livsmedel] [Internet]. Sweden; 2002 [cited 2025 Jul 16]. Available from: https://www.livsmedelsverket.se/globalassets/om-oss/lagstiftning/berikn---kosttillsk---livsm-spec-gr-fsmp/livsfs-2002-19.pdf?siteid:67f9c486-281d-4765-ba72-ba3914739e3b,andquerymatch

19. Sweden. Amendment LIVSFS 2007:9 to Regulation SLVFS 1983:2 on the enrichment of certain foods [Livsmedelsverkets föreskrifter om berikning av vissa livsmedel] [Internet]. 2007 [cited 2025 Jul 18]. Available from: https://www.livsmedelsverket.se/globalassets/om-oss/lagstiftning/berikn---kosttillsk---livsm-spec-gr-fsmp/livsfs-2007-9.pdf

20. Sweden. Swedish Regulation: LIVSFS 2018:5 Regulations on the enrichment of certain foods [Livsmedelsverkets föreskrifter om berikning av vissa livsmedel] [Internet]. 2018. Available from: https://www.livsmedelsverket.se/globalassets/om-oss/lagstiftning/berikn---kosttillsk---livsm-spec-gr-fsmp/livsfs-2018-5_web.pdf

21. Turkey. Turkish Food Codex Regulation on the addition of vitamins and minerals and of certain other substances to foods [TÜRK GIDA KODEKSĠ GIDALARA VĠTAMĠNLER, MĠNERALLER VE BELĠRLĠ  DĠĞER ÖĞELERĠN EKLENMESĠ HAKKINDA YÖNETMELĠK  BĠRĠNCĠ BÖLÜM]. Ministry of Food, Agriculture and Livestock [Internet]. 2017 [cited 2025 Jul 16]. Available from: https://faolex.fao.org/docs/pdf/tur171357.pdf

22. Türkiye. Turkish Food Codex Regulation on the addition of vitamins and minerals and of certain other substances to foods [TÜRK GIDA KODEKSĠ GIDALARA VĠTAMĠNLER, MĠNERALLER VE BELĠRLĠ  DĠĞER ÖĞELERĠN EKLENMESĠ HAKKINDA YÖNETMELĠK  BĠRĠNCĠ BÖLÜM]. Ministry of Food, Agriculture and Livestock [Internet]. 2020 [cited 2025 Jul 18]. Available from: https://faolex.fao.org/docs/pdf/tur196610.pdf

23. United Kingdom. The Bread and Flour Regulations [Internet]. 1998 [cited 2025 Jul 16]. Available from: https://www.legislation.gov.uk/uksi/1998/141/made

24. United Kingdom. The Bread and Flour (Amendment) (England) Regulations [Internet]. 2024 [cited 2025 Jul 16]. Available from: https://www.legislation.gov.uk/uksi/2024/1162/made
